# Estimating different order polynomial logarithmic environmental Kuznets curves

**DOI:** 10.1007/s11356-021-13463-y

**Published:** 2021-04-01

**Authors:** Fakhri J. Hasanov, Lester C. Hunt, Jeyhun I. Mikayilov

**Affiliations:** 1 King Abdullah Petroleum Studies and Research Center (KAPSARC), PO Box 88550, Riyadh, 11672 Saudi Arabia; 2 Economics and Finance, University of Portsmouth, Richmond Building, Portsmouth, PO1 3DE UK

**Keywords:** Environmental Kuznets curve (EKC), Rescaling, Unit dependence, Quadratic, cubic, and quartic specifications, C02, C18, C65, Q51, Q56

## Abstract

This paper contributes to the environmental literature by (i) demonstrating that the estimated coefficients and the statistical significance of the non-leading terms in quadratic, cubic, and quartic logarithmic environmental Kuznets curve (EKC) specifications are arbitrary and should therefore not be used to choose the preferred specification and (ii) detailing a proposed general-to-specific type methodology for choosing the appropriate specifications when attempting to estimate higher-order polynomials such as cubic and quartic logarithmic EKC relationships. Testing for the existence and shape of the well-known EKC phenomenon is a hot topic in the environmental economics literature. The conventional approach widely employs quadratic and cubic specifications and more recently also the quartic specification, where the variables are in logarithmic form. However, it is important that researchers understand whether the estimated EKC coefficients, turning points, and elasticities are statistically acceptable, economically interpretable, and comparable. In addition, it is vital that researchers have a clear structured non-arbitrary methodology for determining the preferred specification and hence shape of the estimated EKC. We therefore show mathematically and empirically the arbitrary nature of estimated non-leading coefficients in quadratic, cubic, and quartic logarithmic EKC specifications, being dependent upon the units of measurement chosen for the independent variables (e.g. dependent upon a rescaling of the variables such as moving from $m to $bn). Consequently, the practice followed in many previously papers, whereby the estimates of the non-leading terms are used in the decision to choose the preferred specification of an estimated EKC relationship, is incorrect and should *not* be followed since it potentially could lead to misleading conclusions. Instead, it should be based upon the sign and statistical significance of the estimated coefficients of the leading terms, the location of turning point(s), and the sign and statistical significance of the estimated elasticities. Furthermore, we suggest that researchers should follow a proposed general-to-specific type methodology for choosing the appropriate order of polynomials when attempting to estimate higher-order polynomial logarithmic EKCs.

**Supplementary Information:**

The online version contains supplementary material available at 10.1007/s11356-021-13463-y.

## Introduction

The pollution-income relationship (PIR) is an important topic that is increasingly investigated by researchers. Since the early work by Grossman and Krueger (1991) and Shafik and Bandyopadhyay (1992), many researchers have attempted to estimate a PIR empirically, based on the idea of an environmental Kuznets curve (EKC) (see, e.g. surveys by Dinda 2004, 2005; Stern 2004; Lieb 2002; and Uchiyama 2016 inter alia). Such studies have attempted to estimate the impact of economic growth on various indicators of environmental degradation, such as carbon dioxide (CO_2_) and sulphur dioxide (SO_2_) emissions, particulate matter, and water pollution.

Kuznets (1955) originally suggested that, as an economy develops, inequality first rises and then decreases—giving rise to the inverted U-shaped relationship known as the Kuznets curve. This concept has since been used in several areas, such as the PIR, where it is hypothesised that as an economy develops, environmental pollution first rises and then decreases—giving rise to what has become known as the inverted U-shaped EKC, first referred to by Panayotou (1993).^1^ Arguably, following the original Kuznets curve, the EKC refers only to a quadratic relationship that produces an inverted U-shaped curve. However, in the environmental economics literature that investigates the PIR to discover whether the EKC hypothesis holds for a given country or group of countries, the general framework tends to now be referred to as the EKC. In other words, although the EKC is regarded as only one special case of the PIR, it is now used in much wider terms to represent any non-linear PIR, whether it be a quadratic specification, a cubic specification, or a quartic specification—or theoretically any *n*th-order polynomial. Therefore, for consistency with the literature, we use the term EKC to represent any non-linear PIR (potentially of any order).

Researchers in this area initially tried to determine whether environmental degradation indicators have an inverted U-shaped relationship with income since such a quadratic relationship would suggest that environmental degradation increases in the early stages of economic growth, before eventually peaking and then decreasing after income reaches a certain level—similar to the original Kuznets (1955) idea for inequality and economic growth, as indicated above. However, as also mentioned above, this area of research has now been extended, with researchers also attempting to determine whether environmental degradation indicators have an N-shaped relationship with income by estimating a cubic relationship or even an M-shaped relationship, with income by estimating a quartic relationship. However, as Destek et al. (2020) demonstrate, a quadratic specification could give a U-shaped EKC, a cubic specification could give an inverted N-shaped EKC, and a quartic specification could give an inverted M-shaped (or W-shaped) EKC. Empirically, these cases, discussed by Destek et al. (2020), might be found with the initial part of the curve being downward sloping. However, a downward-sloping initial part of the curve is inconsistent with the a priori theoretical expectation that the initial stage of any estimated EKC would be upward sloping, which would reflect the initial development stage of the economy or economies being considered and the increasing environmental degradation that occurs during this stage. Therefore, an estimated EKC curve that deviates from an initial upward sloping part warrants further investigation.

As is well known in the environmental economics literature, these non-linear EKCs can be estimated using variables in levels or, as is more often the case, in logarithms, which is this paper’s focus. In particular, we consider the properties of such non-linear EKCs when the variables are in logarithms, showing that the estimated coefficient signs and the statistical significance of the non-leading terms in such specifications are arbitrary, being dependent upon the units of measurement chosen for the independent variables (i.e. being dependent upon different rescaling). We therefore propose a methodology for choosing the appropriate higher-order polynomials for a logarithmic EKC. However, before considering this critical issue, we detail several outstanding issues surrounding the estimation of PIRs/EKCs.

The next section therefore details the outstanding issues in the EKC environmental economics literature followed by Section 3 that initially outlines the functional forms for the quadratic, cubic, and quartic EKC specifications, before focusing on the quadratic logarithmic EKC specification. It provides mathematical proofs of the unit dependence of the coefficients of non-leading terms and the invariance of the coefficients of the leading terms, elasticities, and their *t*-values.^2^ It also shows that the estimated turning points are effectively non-unit dependent. Section 4 follows with an empirical illustration and a discussion of the findings for the estimation of quadratic, cubic, and quartic EKCs. Section 5 summarises and concludes the study. Additionally, for completeness, in an on-line Annex, Appendix 1 details all the current issues around the EKC literature summarised above, and Appendix 2 details the mathematical proofs for the cubic and quartic EKC functional forms.

## Outstanding issues in the EKC environmental economics literature

Although it is almost 30 years since the publication of the Grossman and Krueger (1991) and Shafik and Bandyopadhyay (1992) papers, there are still many unresolved issues in the EKC environmental economics literature. These issues can be broadly grouped into ‘theoretical’, ‘empirical’, and ‘mathematical/statistical’ nuances and are discussed in detail in Appendix 1. In summary, these issues include the following:

- The real-life representativeness of the EKC specification (see Beckerman 1992; Panayotou 1993; Rothman 1998; Mills Busa 2013; Choumert et al. 2013, inter alia)
- The limited capacity of the instruments of environmental indicators (see Schindler 1996, inter alia)
- The use of production- versus consumption-based environmental indicators (see Rothman 1998; Gawande et al. 2001; Bagliani et al. 2008, inter alia)
- Contradicting empirical results (see Roca et al. 2001; Khanna and Plassmann 2004; Auci and Becchetti 2006; Fosten et al. 2012; Yang et al. 2015a; Liddle and Messinis 2016; Mikayilov et al. 2018; Arshad et al. 2020, inter alia)
- The use of per capita versus total income (see Selden and Song 1994; Friedl and Getzner 2003, inter alia)
- The integration-cointegration properties of the used variables (see Stern et al. 1996; Stern 2004; Romero-Avila 2008, inter alia)
- The use of powers of non-stationary variables (see Fürstenberger and Wagner 2007; Wagner 2008, 2012, 2015; Hong and Wagner 2008, inter alia)
- The type of functional specifications used and the econometric techniques employed (see Galeotti et al. 2006; Liddle and Messinis 2016; Apergis 2016; Moosa 2017; Mikayilov et al. 2018, inter alia)
- The use of a trend in the specification, level versus logarithmic variables, and issues related to the turning point (see Lieb 2003; Dinda 2004, inter alia)
- The different cases of the potential relationship between income and environmental degradation, such as monotonic, quadratic (inverted U-shaped), cubic (N-shaped), and quartic (M-shaped) (see Shafik and Bandyopadhyay 1992; Grossman 1995; Lieb 2003, Hasanov et al. 2019, inter alia)

The above summary indicates (and Appendix 1 details) that there are many issues around the modelling of the EKCs, with some way to go before a clear understanding of the environmental quality to economic growth relationship is definitively established. The issues that we focus on in this paper, explained in detail below, are connected in various ways to the issues discussed in the final three bullets of the list above, and, as far as we know, these have not been discussed in the environmental literature before.

Despite all the issues above, the PIR is, in all probability, likely to be a non-linear EKC relationship, which can be rationalised from both theoretical and mathematical/statistical viewpoints. From a theoretical perspective, as mentioned by Lieb (2003) and Dinda (2004) inter alia, there is a demand for environmental quality, regardless of whether it is a normal or a luxury good. At the early stage of a country’s/society’s development, meeting the first items at the top of the demand pyramid is preferred (Maslow 1943), and controlling environmental degradation is not seen as the major concern. Later, when the early necessities are met and the economy is wealthier, environmental awareness and cleaner nature become an increasing concern, likely resulting in a change in the PIR. However, it could also be argued that, due to technological progress and sectoral shifts, after the first turning point, as income continues to rise, a second turning point might emerge giving an N-shaped PIR, or even a second or a third turning point giving an M-shaped PIR (Yang et al. 2015b; Terrell 2020). As mentioned above, some researchers such as Destek et al. (2020) suggest that such non-linear specifications might result in an inverted N-shaped PIR or inverted M-shaped (W-shaped) PIR, depending on the estimates obtained. However, we argue that this is likely due to the initial development stage being missed in the estimation. Either way, given the growing interest in the estimation of quadratic, cubic, and quartic PIRs/EKCs, it would appear prudent to fully understand the properties of such relationships, which is the fundamental issue considered in this paper.

Accepting the argument that the PIR is likely to be non-linear, the issue becomes what is the best specification and what is the appropriate order of the polynomial to capture the non-linearities when attempting to model the EKC. Researchers therefore require a clear understanding of the properties of such specifications and of the interpretation of the results obtained. These are important issues that arguably should be added to the summary list above and ones that, as far as we know, have not been adequately explored in the PIR/EKC environmental literature. Hence, this critical issue is addressed in detail in this paper.

The issue is that the coefficient estimates and their associated *t*-values (and hence their significance levels) in multiplicative-logarithmic functions vary according to the different units of measurement used for expressing the variables, as noted by Hunt and Lynk (1993). Thus, in estimating a logarithmic EKC, the inclusion of a squared term renders the coefficient estimates of the other level term arbitrary and hence meaningless, depending upon the units of measurement chosen for the variables (or, in other words, when the variables are rescaled). Similarly, the inclusion of a cubed term when estimating a logarithmic EKC renders the coefficient estimates of the squared term and the level term arbitrary and hence meaningless when the variables are rescaled. In addition, the inclusion of a term to the power of 4 when estimating a logarithmic EKC renders the coefficient estimates of the cubed term, the squared term, and the level term arbitrary and hence meaningless when the variables are rescaled. As stated, as far as we are aware, this issue has not been considered in the environmental economics literature. As highlighted later in the paper, many previous EKC studies have chosen the preferred specification based on these arbitrary and meaningless estimates—hence this is a cautionary tale given that many previous studies might have potentially accepted inappropriate results. Moreover, given this problem, we offer a way forward, suggesting criteria that researchers should adopt when determining the existence and shape of an estimated logarithmic EKC.

## Properties of the logarithmic EKC

### Background of the logarithmic EKC specification

The many attempts to estimate an EKC relationship have used a variety of specifications with both time series and panel data. Later in this section, for expositional reasons, we focus on the quadratic version, but first we outline the three relatively popular versions of the EKC. Letting *E*_*t*_ = environmental degradation (pollution) per capita^3^ in time *t*, *Y*_*t*_ = income or GDP per capita in time *t*,^4^*ln* = natural logarithm, and the *α*’s and *β*’s parameters to be estimated, the base specifications of the non-linear EKC, namely the quadratic, cubic, and quartic forms, are as follows:^5^

*Linear (not in logarithmic form)*

1a $$ {E}_t={\alpha}_0+{\alpha}_1{Y}_t+{\alpha}_2{Y}_t^2 $$

1b $$ {E}_t={\alpha}_0+{\alpha}_1{Y}_t+{\alpha}_2{Y}_t^2+{\alpha}_3{Y}_t^3 $$

1c $$ {E}_t={\alpha}_0+{\alpha}_1{Y}_t+{\alpha}_2{Y}_t^2+{\alpha}_3{Y}_t^3+{\alpha}_4{Y}_t^4 $$

*Logarithmic* 6

2a $$ \mathit{\ln}{E}_t={\beta}_0+{\beta}_1\mathit{\ln}{Y}_t+{\beta}_2{\mathit{\ln}}^2{Y}_t $$

2b $$ \mathit{\ln}{E}_t={\beta}_0+{\beta}_1\mathit{\ln}{Y}_t+{\beta}_2{\mathit{\ln}}^2{Y}_t+{\beta}_3{\mathit{\ln}}^3{Y}_t $$

2c $$ \mathit{\ln}{E}_t={\beta}_0+{\beta}_1\mathit{\ln}{Y}_t+{\beta}_2{\mathit{\ln}}^2{Y}_t+{\beta}_3{\mathit{\ln}}^3{Y}_t+{\beta}_4{\mathit{\ln}}^4{Y}_t $$

Attempts have been made to estimate an EKC using all specifications shown in Eqs. (1) and (2).^7^ For example, Shafik and Bandyopadhyay (1992) estimated linear, quadratic, and cubic models in logs; Grossman and Krueger (1995) estimated cubic model in levels (without logs); Harbaugh et al. (2002) estimated cubic specification in logs as well as in levels; Lieb (2002) estimated cubic specification in logs; Ang (2007), Shahbaz et al. (2014), Onafowora and Owoye (2014), Alshehry and Belloumi (2016), and many others estimated quadratic models in logs; Yang et al. (2015a) used all three functional forms in logs (linear, quadratic, and cubic); Li et al. (2019) estimated quadratic and cubic models in logs; Yang et al. (2015b) estimated all polynomial functions (in logs) from the first to the fifth order; and Terrell (2020) estimated quartic specification in both levels and logs.

However, as far as we are aware, there has been no discussion in the PIR/EKC literature about the effect of using different units of measurement for the main explanatory variable, income (such as moving from $bn to $m), and how to determine the appropriate preferred polynomial specification. For the functional specifications given by Eqs. (1a), (1b), and (1c) in levels, it is known that for *α*_1_ , *α*_2_, *α*_3_, and *α*_4_, the sizes (which do not affect any decision about the shape) are unit dependent, while the signs and significances are unit independent. This means that the coefficients can be used to decide the curvature/shape of the relationship. However, Hunt and Lynk (1993) showed that in multiplicative-logarithmic functions other than for the coefficient of the highest power in the polynomial function (hereafter referred to as the leading term), the estimated coefficients and their significances of the lower power terms are sensitive to rescaling (i.e., *unit dependent*), whereas the elasticities are not sensitive to rescaling (i.e., *unit invariant*).^8^ Given this, Hunt and Lynk (1993) suggest that the focus of reporting and interpretation should be on the leading terms and the estimated elasticities, rather than the lower power coefficients. This issue is therefore explored here, since in conventional PIR/EKC studies, the preferred models have often been chosen based upon both the individual estimated coefficient signs and significance. This has been the case in many papers published on estimating an EKC that have been included in several leading environmental economics journals. For example, these include, for the common quadratic specification like Eq. (2a), the following:

- In *Energy Policy*, Ang (2007; p. 4774) states ‘Under the EKC hypothesis, [β_1_] is expected to be positive whereas a negative sign is expected for [β_2_]’.
- In *Sustainable Development*, Atici (2008; p. 158) states that according ‘to the EKC, we may expect the sign of β_1_ to be positive and that of β_2_ to be negative’.
- In *Energy*, Tang and Tan (2015; p. 449) state ‘According to the EKC hypothesis, the sign of β_1_ is expected to be positive, while the sign of β_2_ is expected to be negative’.
- In *Economic Modelling*, Kasman and Duman (2015; p. 98) state ‘Under the EKC hypothesis, it is expected that β_1_ > 0 and if β_2_ < 0. Hence, there is an inverted U-shaped pattern’.
- In *Renewable and Sustainable Energy Reviews*, Jebli and Youssef (2015; p. 178) state ‘Under the EKC hypothesis, the sign of [β_1_] is expected to be positive, whereas [β_2_] is expected to be negative’.
- In *Ecological Indicators*, Al-Mulali et al. (2016; p. 274) state ‘The existence of the EKC hypothesis, which indicates the inverted U-shaped relationship between income and CO_2_ emission, can be confirmed if β_1_ > 0 … and β_2_ < 0’.
- In *Ecological Economics*, Bimonte and Stabile (2017; pp. 39-40) state ‘Contrary to expectations, the main stylized fact … is that the relationship between per capita income and conservation of environmental resources … follows a U-shaped path (β_1_ < 0; β_2_ > 0’).^9^
- In *Energy Economics*, Balaguer and Cantavella (2018; p. 290) state “Under the assumption of a conventional EKC we expect that [β_1_ > 0] and [β_2_ < 0];
- In *Renewable Energy*, Sharif et al. (2019; p. 689) state “It can be seen from the results … that the economic growth has a positive value whereas the square rate of economic growth shows the negative value. This confirms the existence of the Kuznets curve hypothesis i.e., the inverted U-shape association between the economic growth and CO2 emission”;
- In *Structural Change and Economic Dynamics,* Jiang et al. (2019; p. 248) state if “ … [β_1_ > 0] and [β_2_ < 0], it reveals an inverted U-shaped curve” but if “… [β_1_ < 0] and [β_2_ > 0], it indicates a U-shaped curve”; and
- In *Environmental Science and Pollution Research*, Gormus and Aydin (2020; p. 27908) state “… the coefficient of [lnY_t_] should be positive and the coefficient of [ln^2^Y_t_] should be negative…”

This list represents just a few examples of where researchers have made such statements when estimating a quadratic EKC using logarithms. In addition, some authors have made similar statements when attempting to estimate a cubic EKC like Eq. (2b), such as the following:

- In *Energy Economics*, Baek (2015; p. 14) state ‘… known as an N-shape curve, it is expected that β_1_ >0, β_2_ < 0 and β_3_ >0’.
- In *Economics of Energy & Environmental Policy*, Sorge and Neumann (2020; p. 174) state ‘β_1_, β_2_, and β_3_ are statistically significant and β_1_ >0, β_2_ < 0 and β_3_ >0, which … suggests a N-shaped pattern’.

Other examples include Yang et al. (2015a, b), Jaforullah and King (2017); Terrell (2020), and Li et al. (2019). However, as we show below, such statements are misleading for the logarithmic versions of the EKC shown in Eq. (2), since the size, sign, and significance of the non-leading terms are irrelevant to the decision of whether the inverted U-shaped, N-shaped, or M-shaped EKC exists or not. Therefore, as stated, the remainder of this section focuses on the quadratic specification, Eq. (2a), for ease of exposition and because it is the most popular specification in the environmental economics literature. Nonetheless, the issue is equally important for researchers attempting to estimate cubic or quartic PIRs/EKCs (which are increasing in the literature), but the theoretical results for these can easily be generalised for the cubic and quartic cases (and are detailed in Appendix 2 for completeness). Moreover, in Section 4 we present examples of empirical estimates for all cases and develop a structured methodology for choosing the preferred order of polynomial for the estimated logarithmic EKC.

### Some algebra for the quadratic logarithmic EKC

As discussed above, the quadratic version of the EKC is more popular in the literature, whether after ‘testing down’ or by assumption; hence, as indicated above, we focus here in the algebra section on Eq. (2a). Using the definitions above, the ‘raw’ data for *Y*_*t*_ could be re-based by multiplying by an arbitrary constant (e.g. to convert the ‘raw’ data to millions or thousands or to index to a certain base year). Therefore, letting *a* be the rescaling factor, the new independent variable becomes:

3 $$ {Y}_t^{\ast }=a{Y}_t $$

so that Eq. (2a) becomes:

4 $$ \mathit{\ln}{E}_t={\beta}_0^{\ast }+{\beta}_1^{\ast}\mathit{\ln}{Y}_t^{\ast }+{\beta}_2^{\ast }{\mathit{\ln}}^2{Y}_t^{\ast } $$

#### Estimated parameters and statistical significance

After substituting Eq. (3) into Eq. (4) and re-arranging, it becomes:

5 $$ \mathit{\ln}{E}_t=\left[{\beta}_0^{\ast }+{\beta}_1^{\ast } lna+{\beta}_2^{\ast }{\mathit{\ln}}^2a\right]+\left[{\beta}_1^{\ast }+2{\beta}_2^{\ast } lna\right]\mathit{\ln}{Y}_t+{\beta}_2^{\ast }{\mathit{\ln}}^2{Y}_t $$

Given that the left-hand side of Eq. (2a) and Eq. (5) are the same, we can equate the right-hand sides to relate the previous and new coefficients so that:

6a $$ {\beta}_0^{\ast }={\beta}_0-{\beta}_1 lna+{\beta}_2{\mathit{\ln}}^2a $$

6b $$ {\beta}_1^{\ast }={\beta}_1-2{\beta}_2 lna $$

6c $$ {\beta}_2^{\ast }={\beta}_2 $$

In this case, Eq. (6c) shows that the coefficient on the quadratic (leading) term is invariant to the units of measurement, while the other coefficients are unit dependent^10^—shown in Eqs. (6a) and (6b).

The natural question to follow this is: Does the rescaling of the variables also affect the statistical significance of the coefficients? To investigate this, we need to consider the effect of the rescaling on the *t*-values of *β*_1_ and *β*_2_, which are defined as follows:

7a $$ {t}_{\beta_1}=\frac{\beta_1}{\sqrt{\mathit{\operatorname{var}}\left({\beta}_1\right)}} $$

7b $$ {t}_{\beta_2}=\frac{\beta_2}{\sqrt{\mathit{\operatorname{var}}\left({\beta}_2\right)}} $$

7c $$ {t}_{\beta_1^{\ast }}=\frac{\beta_1^{\ast }}{\sqrt{\mathit{\operatorname{var}}\left({\beta}_1^{\ast}\right)}} $$

7d $$ {t}_{\beta_2^{\ast }}=\frac{\beta_2^{\ast }}{\sqrt{\mathit{\operatorname{var}}\left({\beta}_2^{\ast}\right)}} $$

Substituting Eqs. (6b) and (6c) into Eqs. (7c) and (7d) gives:

8a $$ {t}_{\beta_1^{\ast }}=\frac{\beta_1-2{\beta}_2 lna}{\sqrt{\mathit{\operatorname{var}}\left({\beta}_1-2{\beta}_2 lna\right)}}=\frac{\beta_1}{\sqrt{\mathit{\operatorname{var}}\left({\beta}_1\right)}}\sqrt{\frac{\ \mathit{\operatorname{var}}\left({\beta}_1\right)}{\ \mathit{\operatorname{var}}\left({\beta}_1-2{\beta}_2 lna\right)}}-2 lna\frac{\beta_2}{\sqrt{\mathit{\operatorname{var}}\left({\beta}_2\right)}}\sqrt{\frac{\ \mathit{\operatorname{var}}\left({\beta}_2\right)}{\ \mathit{\operatorname{var}}\left({\beta}_1-2{\beta}_2 lna\right)}} $$

and

8b $$ {t}_{\beta_2^{\ast }}=\frac{\beta_2}{\sqrt{\mathit{\operatorname{var}}\left({\beta}_2\right)}} $$

Therefore, comparing Eq. (7a) with Eq. (8a) and Eq. (7b) with Eq. (8b) shows that:

9a $$ {t}_{\beta_1}\ne {t}_{\beta_1^{\ast }} $$

whereas

9b $$ {t}_{\beta_2}={t}_{\beta_2^{\ast }} $$

Thus, the statistical significance of the coefficient of the non-leading term (like its sign and size) is *unit dependent* given that the *t*-values will vary according to different scaling, whereas the statistical significance of the coefficient of the leading term (like its sign and size) is *unit invariant*, given the *t*-value does not vary due to different scaling. This implies that the only *necessary condition* for a quadratic (inverted U-shaped) EKC is that the leading term *β*_2_ is negative and statistically significant.^11^ The necessary conditions for the cubic (N-shaped) and quartic (M-shaped) EKCs are detailed in Appendix 2.

#### Estimated turning point

The turning point for the quadratic logarithmic EKC specification, Eq. (2a), is found by differentiating with respect to Y and setting equal to zero. This is given by^12^:

10a $$ {Y}^{TP}=\exp \left(\frac{-{\beta}_1}{2{\beta}_2}\right) $$

but for Eq. (4) with the re-based units, this gives:

10b $$ {Y}^{\ast TP}=\exp \left(\frac{-{\beta}_1^{\ast }}{2{\beta}_2^{\ast }}\right) $$

However, substituting Eqs. (6b) to (6c) into Eq. (10b) gives:

10c $$ {\displaystyle \begin{array}{c}{Y}^{\ast TP}=\exp \left(-\frac{\beta_1-2{\beta}_2 lna}{2{\beta}_2}\right)=\exp \left(-\frac{\beta_1}{2{\beta}_2}+ lna\right)\\ {}=\exp \left(-\frac{\beta_1}{2{\beta}_2}\right)\exp (lna)=a\exp \left(-\frac{\beta_1}{2{\beta}_2}\right)=a{Y}^{TP}\end{array}} $$

Therefore, this shows that the rescaling of the income variable results in the turning point of the estimated EKC being rescaled by the same factor.^13^ In other words, as would be expected intuitively, the estimated turning point is effectively the same.

#### Estimated elasticity and statistical significance

Given that we have shown above that the sign, size, and statistical significance of the non-leading term of a quadratic EKC in logarithmic form are unit dependent, we next consider the elasticity of *E* with respect to *Y* for Eq. (2a), which is given by:

11a $$ \eta =\frac{\partial {E}_t}{\partial {Y}_t}\frac{Y_t}{E_t}=\frac{\partial \mathit{\ln}{E}_t}{\partial \mathit{\ln}{Y}_t}={\beta}_1+2{\beta}_2\mathit{\ln}{Y}_t\kern0.5em $$

And the elasticity for the rescaled version, Eq. (4), is given by:

11b $$ {\eta}^{\ast }=\frac{\partial {E}_t}{\partial {Y}_t^{\ast }}\frac{Y_t^{\ast }}{E_t}=\frac{\partial \mathit{\ln}{E}_t}{\partial \mathit{\ln}{Y}_t^{\ast }}={\beta}_1^{\ast }+2{\beta}_2^{\ast}\mathit{\ln}{Y}_t^{\ast } $$

But substituting Eqs. (3), (6b), and (6c) into Eq. (11b) and re-arranging gives:

11c $$ {\eta}^{\ast }={\beta}_1-2{\beta}_2l\mathrm{n}a+2{\beta}_2\mathit{\ln}{Y}_t+2{\beta}_2 lna={\beta}_1+2{\beta}_2\mathit{\ln}{Y}_t $$

so that the elasticity of *E* with respect to *Y* is unit invariant, since as Eqs. (11a) and (11c) show:

11d $$ {\eta}^{\ast }=\eta $$

Furthermore, the statistical significance of the elasticity is also unit independent, since the *t*-values of *η* and *η*^∗^ are identical. To show this, the variance of *η* can be expressed as follows:

12b $$ \mathit{\operatorname{var}}\left(\eta \right)=\mathit{\operatorname{var}}\left({\beta}_1+2{\beta}_2\mathit{\ln}{Y}_t\right)=\mathit{\operatorname{var}}\left({\beta}_1\right)+4\mathit{\ln}{Y}_t\ast \mathit{\operatorname{cov}}\left({\beta}_1,{\beta}_2\right)+4{\mathit{\ln}}^2{Y}_t\ast \mathit{\operatorname{var}}\left({\beta}_2\right) $$

While for *η*^∗^, the variance can be expressed as follows:

12b $$ \mathit{\operatorname{var}}\left({\eta}^{\ast }\ \right)=\mathit{\operatorname{var}}\left({\beta}_1^{\ast }+2{\beta}_2^{\ast}\mathit{\ln}{Y}_t^{\ast}\right) $$

Introducing the rescaling in Eq. (3), and utilising the standard properties of variance and covariance,^14^ Eq. (12b) can be expressed as follows:

12c $$ \mathit{\operatorname{var}}\left({\eta}^{\ast }\ \right)=\mathit{\operatorname{var}}\left({\beta}_1^{\ast }+2{\beta}_2^{\ast}\mathit{\ln}{Y}_t^{\ast}\right)=\mathit{\operatorname{var}}\Big({\beta}_1^{\ast }+2{\beta}_2^{\ast}\ln \left(a{Y}_t\right)=\mathit{\operatorname{var}}\left({\beta}_1^{\ast}\right)+4\ln \left(a{Y}_t\right)\ast \mathit{\operatorname{cov}}\left({\beta}_1^{\ast },{\beta}_2^{\ast}\right)+4{\mathit{\ln}}^2\left({aY}_t\right)\ast \mathit{\operatorname{var}}\left({\beta}_2^{\ast}\right)=\mathit{\operatorname{var}}\left({\beta}_1-2{\beta}_2 lna\right)+4\mathit{\operatorname{cov}}\left({\beta}_1-2{\beta}_2 lna,{\beta}_2\right)\left( lna+\mathit{\ln}{Y}_t\right)+4\ast \mathit{\operatorname{var}}\left({\beta}_2\right){\left( lna+\mathit{\ln}{Y}_t\right)}^2=\mathit{\operatorname{var}}\left({\beta}_1\right)-4 lna\ast \mathit{\operatorname{cov}}\left({\beta}_1,{\beta}_2\right)+4{\mathit{\ln}}^2a\ast \mathit{\operatorname{var}}\left({\beta}_2\right)+4 lna\ast \mathit{\operatorname{cov}}\left({\beta}_1,{\beta}_2\right)-8{\mathit{\ln}}^2a\ast \mathit{\operatorname{var}}\left({\beta}_2\right)+4\mathit{\ln}{Y}_t\ast \mathit{\operatorname{cov}}\left({\beta}_1,{\beta}_2\right)-8 lna\ast \mathit{\ln}{Y}_t\ast \mathit{\operatorname{var}}\left({\beta}_2\right)+4{\mathit{\ln}}^2a\ast \mathit{\operatorname{var}}\left({\beta}_2\right)+8 lna\ast \mathit{\ln}{Y}_t\ast \mathit{\operatorname{var}}\left({\beta}_2\right)+4{\mathit{\ln}}^2{Y}_t\ast \mathit{\operatorname{var}}\left({\beta}_2\right)=\mathit{\operatorname{var}}\left({\beta}_1\right)+4\mathit{\ln}{Y}_t\ast \mathit{\operatorname{cov}}\left({\beta}_1,{\beta}_2\right)+4{\mathit{\ln}}^2{Y}_t\ast \mathit{\operatorname{var}}\left({\beta}_2\right)=\mathit{\operatorname{var}}\left(\eta \right) $$

Eq. (11d) and Eq. (12c) show that the elasticity estimates and their variances are not affected by the rescaling^15^. Hence, their standard errors, *t*-values, and significance levels are all *not unit dependent*.

#### Sufficient condition

Mathematically, conditions are formalised as being ‘necessary’ and ‘sufficient’. As discussed above, the necessary condition for an estimated quadratic (inverted U-shaped) EKC is that the estimated leading term, *β*_2_, is negative (and statistically significant). Mathematically, the sufficient condition for a quadratic (inverted U-shaped) EKC is that $-\frac{\beta_1}{2{\beta}_2}$^16^ is a real number, which holds when *β*_2_ < 0 (and statistically significant). Hence, the sufficient and necessary conditions for a quadratic (inverted U-shaped) EKC are effectively the same. Furthermore, the turning point needs to be within the data range of the sample. In other words, the sufficient condition for an estimated quadratic (inverted U-shaped) EKC, as in Eq. (2a), is that the turning point is within the sample range and the estimated pairwise elasticities are positive and significant for the initial upward sloping part of the estimated curve but they approach zero and become insignificant at the first turning point, thereafter becoming negative and significant on the downward sloping part (similar sufficient conditions for the cubic and quartic EKCs are detailed in Appendix 2).

#### Summary

In this section, we have shown mathematically that for the quadratic logarithmic EKC, the signs, sizes, and significances of the non-leading coefficients are unit dependent, whereas the signs, sizes, and significances of the leading squared term and the estimated elasticity are unit independent. Furthermore, we have also shown mathematically that the estimated turning point, although rescaled, is effectively the same and thus not actually dependent upon the units of measurement of the variables. Although this illustration is for the quadratic EKC specification, the results are easily generalised for the cubic and quartic specifications (as shown in Appendix 2). The next section highlights this by presenting some empirical illustrations for all three specifications.

## Empirical Illustration

This section empirically illustrates the findings from the previous section by employing the Baek (2015) data used by Jaforullah and King (2017) to estimate a CO_2_ emissions EKC for Denmark and Sweden, respectively.^17^ For simplicity, we use ordinary least squares (OLS) to estimate the different EKC specifications. Nonetheless, we realise that more sophisticated methodologies have been, and in all likelihood would be, employed, but the different estimation methods do not change the key messages from this paper.^18^ The next three sub-sections therefore give illustrations for the quadratic, cubic, and quartic specifications, respectively, highlighting the mathematical findings from the previous section and developing an estimation strategy for developing logarithmic EKCs.

### Empirical illustration of the quadratic logarithmic EKC

The results of the empirical illustrations are given in Table 1, showing the estimation results with different scaling factors given in different columns. This shows that the empirical results confirm the finding of the mathematical derivations above. For Denmark, $\hat{\beta_2}=-1.42$with an associated *t*-value at −9.62; both clearly do not change by rescaling the income variable. Similarly, for Sweden, $\hat{\beta_2}=-1.16$ and the associated *t*-value is −4.83. In other words, the sign, size, and statistical significance of this estimated coefficient $\left(\hat{\beta_2}\right)$ are *unit invariant*.

**Table 1:** Quadratic specification estimation results

**Denmark**							
Scaling factor (*a*)	0.00003	0.000032	0.001	1 (original data)	1000	31250	33333
$\hat{\beta_1}$	−0.1976^***^	−0.0138	9.7856^***^	29.4519^***^	49.1182^***^	58.9176^***^	59.1014^***^
*t*-value	(−4.0804)	(−0.3262)	(9.6501)	(9.6372)	(9.6337)	(9.6328)	(9.6328)
$\hat{\beta_2}$	−1.4235^***^	−1.4235^***^	−1.4235^***^	−1.4235^***^	−1.4235^***^	−1.4235^***^	−1.4235^***^
*t*-value	(−9.6278)	(−9.6278)	(−9.6278)	(−9.6278)	(−9.6278)	(−9.6278)	(−9.6278)
$\hat{\eta}$	−0.2375^***^	−0.2375^***^	−0.2375^***^	−0.2375^***^	−0.2375^***^	−0.2375^***^	−0.2375^***^
*t*-value	(−4.7027)	(−4.7027)	(−4.7027)	(−4.7027)	(−4.7027)	(−4.7027)	(−4.7027)
**Swede**n							
Scaling factor (*a*)	0.00003	0.000049	0.001	1 (original data)	1000	20408	33333
$\hat{\beta_1}$	−1.0751***	0.0670	7.0877^***^	23.1680 ^***^	39.2484^***^	46.2690^***^	47.4111^***^
*t*-value	(−8.0723)	(0.4533)	(4.4775)	(4.7162)	(4.7614)	(4.7714)	(4.7728)
$\hat{\beta_2}$	−1.1639^***^	−1.1639^***^	−1.1639^***^	−1.1639^***^	−1.1639^***^	−1.1639^***^	−1.1639^***^
*t*-value	(−4.8278)	(−4.8278)	(−4.8278)	(−4.8278)	(−4.8278)	(−4.8278)	(−4.8278)
$\hat{\eta}$	−0.7025^***^	−0.7025^***^	−0.7025^***^	−0.7025^***^	−0.7025^***^	−0.7025^***^	−0.7025^***^
*t*-value	(−8.6113)	(−8.6113)	(−8.6113)	(−8.6113)	(−8.6113)	(−8.6113)	(−8.6113)

Source: Data from Jaforullah and King (2017)

Notes: The values of the scaling factors are specifically chosen to fully illustrate the possibilities for the estimated coefficients to have opposite signs as well as being supposedly statistically ‘significant’ or ‘insignificant’ (according to *t*-values). The estimated elasticities $\hat{\Big(\eta}\Big)$ are calculated at their mean values, as discussed in Gujarati and Porter (2009), inter alia

**Fig. 1:**
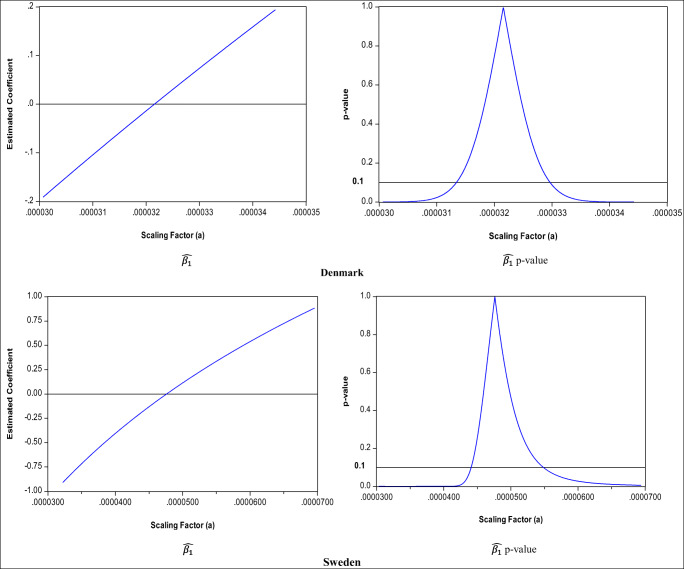
Various estimates for $\hat{\beta_1}$ and associated significance levels for quadratic EKC

However, this is not the case for $\hat{\beta_1}$. Table 1 clearly shows that both the size and the sign for both Denmark and Sweden vary according to the different rescaling, which is further shown in Fig. 1, which gives examples of scaling factors to illustrate the possibility of estimated coefficients with opposite signs and with significant and insignificant values (according to *t*-values). Figure 1 therefore shows that there is a range of scaling factors whereby the estimates of $\hat{\beta_1}$ would be negative and another range that would suggest that $\hat{\beta_1}$is not significantly different from zero. As can be seen from Table 1, for Denmark, when the scaling factor is 0.00003, $\hat{\beta_1}$ is negative, and for the last five scaling factors, it is positive. Furthermore, when the scaling factor is 0.000032, $\hat{\beta_1}$ is also statistically insignificant. For Sweden, $\hat{\beta_1}$ is negative when the scaling factor is 0.00003 and statistically insignificant when it is 0.000049. In short, Table 1 and Fig. 1 illustrate that, for both countries, $\hat{\beta_1}$ can take negative or positive values as well as zero (e.g. the first column in Table 1). It also shows that there is a range of scaling factors (illustrated by the horizontal 10% significance line in Fig. 1) for which the estimated coefficients become statistically insignificant (e.g. the second column in Table 1). This clearly shows that the sign, size, and statistical significance of the coefficient $\hat{\beta_1}$ are *unit dependent*.

**Fig. 2:**
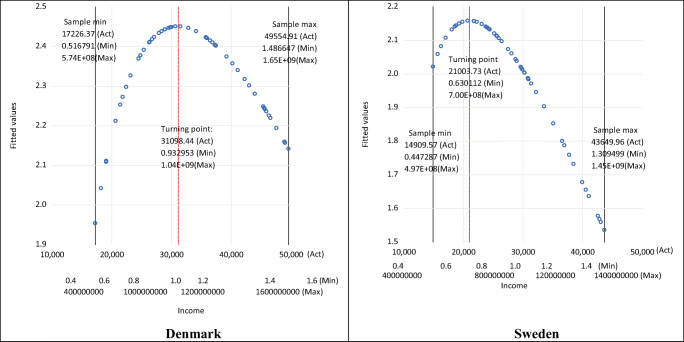
Estimated turning points with scaled per capita income. (Note: Act, *a* =1; Min, *a* =0.00003; Max, *a* =33333. These represent the examples of the scaling factors shown on the income axes.)

Figure 2 illustrates the estimated shapes of the EKCs and the turning points for three different scaling factors: *a*—labelled as ‘Act’ for the actual original data (where *a* =1), ‘Min’ for the smallest scaling factor used in the chart, and ‘Max’ for the largest scaling factor used in the chart. Note, in Fig. 2 the three different, relatively extreme, values for the income scaling factor *a* shown for the income axis are chosen for illustrative purposes only and highlight that the same results would apply for CO_2_ emissions whatever scaling factor were used. Figure 2 shows, as detailed above, that the estimated EKC turning point for both Denmark and Sweden is scaled by the same scaling factor used to scale the independent variable in functional form Eq. (2a). Thus, the shape of the EKCs for each country is identical, and the turning points in terms of emissions are the same, but with the income just scaled accordingly. In other words, $\hat{\beta_1}$ does *not* have an impact on the actual shape of the estimated EKC and where the important turning point is—whether $\hat{\beta_1}$ is positive or negative and/or statistically significant or insignificant. Therefore, decisions by researchers on the acceptance or otherwise the existence and shape of an estimated EKC should *not* be based on $\hat{\beta_1}$.

**Fig. 3:**
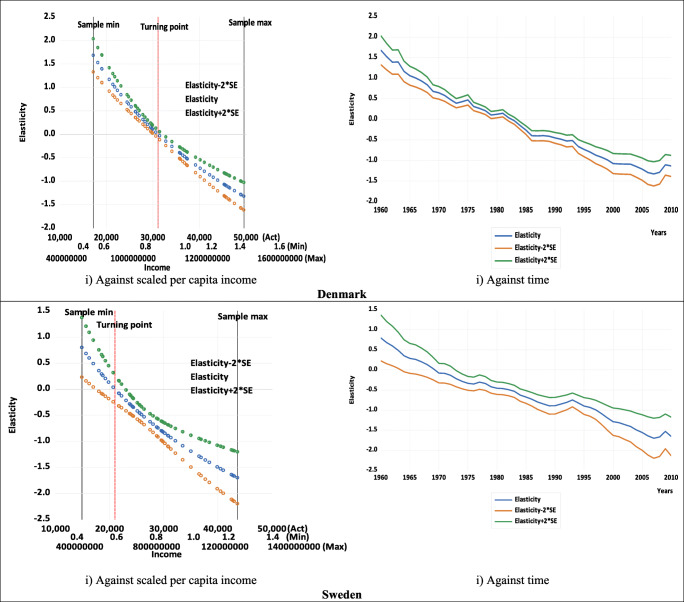
Estimated pointwise elasticities with 95% confidence intervals. (Note: Act, *a* =1; Min, *a* =0.00003; Max, *a* =33333. These represent the examples of the scaling factors shown on the income axes.)

Table 1 also shows that, for both countries, the estimated summary elasticities (calculated at mean values as discussed in Gujarati and Porter 2009), as well as their *t*-values (and thus their statistical significance) are invariant to rescaling. Thus, when deciding on the acceptance or otherwise the existence and shape of an estimated logarithmic EKC, there should be greater focus on the estimated elasticity and not the estimate of *β*_1_. In other words, since the estimate of *β*_1_ is unit variant in terms of sign and significance, it does not provide any useful information about the shape of the relationship.^19^ Instead, for an inverted U-shaped EKC to hold, one of the necessary conditions is having a statistically significant elasticity that is positive before the turning point and becomes negative after the turning point (or at least for some sample values). Figure 3 illustrates the estimated pointwise elasticities for both countries, both against income and time, using the examples of three different scaling factors *a*. These clearly show that the elasticity estimates are not unit dependent (both when plotted against income and time). Figure 3 also shows that, for the quadratic case, the turning points for both countries are within the sample size, which is important when deciding upon the preferred specification—an issue we will return to later as we explore the cubic and quartic cases.

The charts plotted against income in Fig. 3 also show that for both countries, the estimated pointwise elasticities are positive and significant at low levels of income but gradually fall, becoming insignificant the closer to the estimated turning point, where the elasticity would be zero. Thereafter, as income increases, the estimated elasticities continue to fall, gradually becoming more negative and significant. The charts against time in Fig. 3 show that for both countries, the estimated pointwise elasticities are initially positive and fall over time and then become increasingly negative.

### Empirical illustration of the cubic logarithmic EKC

**Fig. 4:**
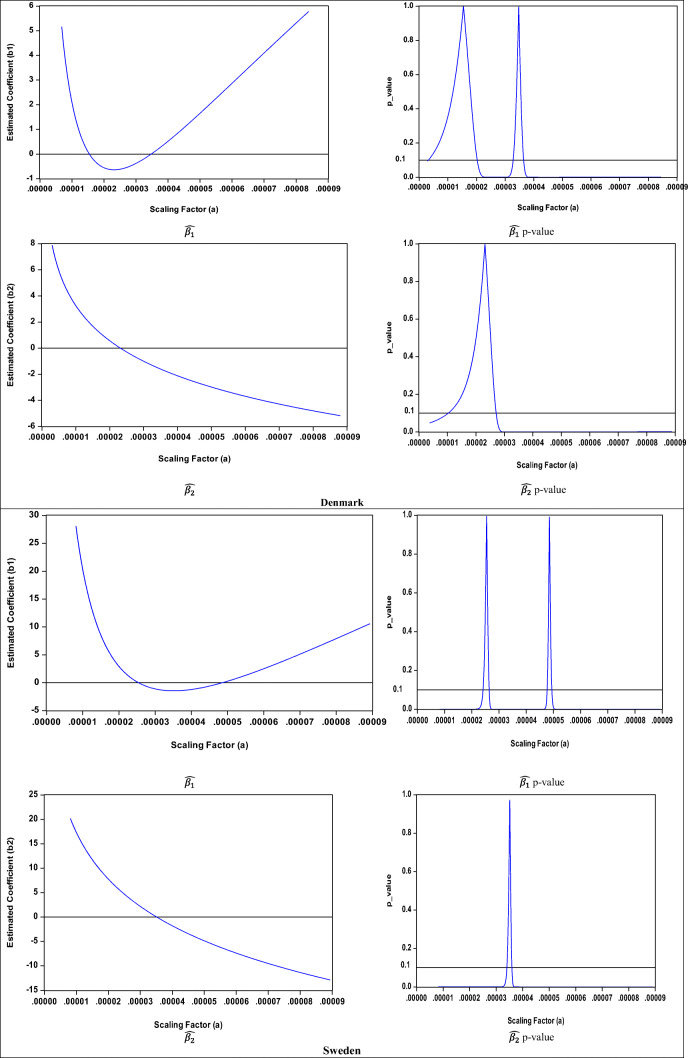
Various estimates for $\hat{\beta_1}$ and $\hat{\beta_2}$ and associated significance levels for cubic EKC

The results for the empirical estimation for the cubic case are presented in Table 2 and illustrated in Fig. 4. These show that the cubic specification results display the same issue as the quadratic case. Although the size, sign, and significance of the estimated coefficients of the leading term ($\hat{\beta_3}$) are unit independent, this is not the case for $\hat{\beta_1}$ and $\hat{\beta_2}$ since Table 2 and Fig. 4 clearly show that, for all the illustrative examples, their sign, size, and significance are unit dependent. Moreover, there is a range of scaling factors (illustrated by the horizontal 10% significance line in the second column of Fig. 4) for which the coefficients $\hat{\beta_1}$ and $\hat{\beta_2}$ become statistically insignificant. Furthermore, like the quadratic case, the estimated summary elasticities and their *t*-values (and thus their statistical significance) are invariant to rescaling.

**Table 2:** Cubic specification estimation results

**Denmark**							
Scaling factor (*a*)	0.000017	0.001	0.01	1 (original data)	10	100	1000
$\hat{\beta_1}$	−0.2640	54.2493^**^	141.9168^**^	440.4372^**^	651.2899^**^	903.2044^**^	1196.1807^**^
*t*-value	(−0.3986)	(2.9750)	(2.7583)	(2.6165)	(2.5845)	(2.5625)	(2.5463)
$\hat{\beta_2}$	1.1996	−14.5786^***^	−23.4950^***^	−41.3279^***^	−50.2443^***^	−59.1608^***^	−68.0772^***^
*t*-value	(1.1072)	(−2.7052)	(−2.5990)	(−2.5289)	(−2.5130)	(−2.5020)	(−2.4940)
$\hat{\beta_3}$	1.2908^***^	1.2908^***^	1.2908^***^	1.2908^***^	1.2908^***^	1.2908^***^	1.2908^***^
*t*-value	(2.4419)	(2.4419)	(2.4419)	(2.4419)	(2.4419)	(2.4419)	(2.4419)
$\hat{\eta}$	−0.4047^***^	−0.4047^***^	−0.4047^***^	−0.4047^***^	−0.4047^***^	−0.4047^***^	−0.4047^***^
*t*-value	(−4.8716)	(−4.8716)	(−4.8716)	(−4.8716)	(−4.8716)	(−4.8716)	(−4.8716)
**Sweden**							
Scaling factor (*a*)	0.000035	0.0000483	0.001	0.01	1 (original data)	100	1000
$\hat{\beta_1}$	−1.4409^***^	−0.0417	153.3242^***^	439.3269^***^	1450.3580^***^	3046.7567^***^	4064.4689^***^
*t*-value	(−13.4025)	(−0.4458)	(8.1970)	(8.0558)	(7.9585)	(7.9205)	(7.9091)
$\hat{\beta_2}$	0.0504	−4.3946^***^	−46.2158^***^	−77.9936^***^	−141.5490^***^	−205.1045^***^	−236.8823^***^
*t*-value	(0.2275)	(−9.9483)	(−8.0294)	(−7.9493)	(−7.8973)	(−7.8776)	(-7.8717)
$\hat{\beta_3}$	4.6003^***^	4.6003^***^	4.6003^***^	4.6003^***^	4.6003^***^	4.6003^***^	4.6003^***^
*t*-value	(7.8326)	(7.8326)	(7.8326)	(7.8326)	(7.8326)	(7.8326)	(7.8326)
$\hat{\eta}$	−1.4410^***^	−1.4410^***^	−1.4410^***^	−1.4410^***^	−1.4410^***^	−1.4410^***^	−1.4410^***^
*t*-value	(−13.1143)	(−13.1143)	(−13.1143)	(−13.1143)	(−13.1143)	(−13.1143)	(−13.1143)

Source: Data from Jaforullah and King (2017)

Notes: The values of the scaling factors are chosen to show the existence of coefficients with the opposite signs and with significant and insignificant values (according to *t*-values). The estimated elasticities $\hat{\Big(\eta}\Big)$ are calculated at mean values as discussed in Gujarati and Porter (2009), inter alia

**Fig. 5:**
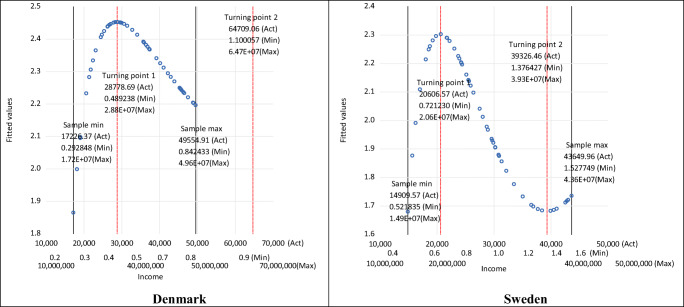
Estimated turning points with scaled per capita income. (Note: For Denmark, Act, *a* =1; Min, *a* =0.000017; Max, *a* =1000; for Sweden, Act, *a* =1; Min, *a* =0.000035; Max, *a* =1000. These represent the examples of the scaling factors shown on the Denmark and Sweden income axes, respectively.)

Like Fig. 2 for the quadratic specification, Fig. 5 shows for the cubic specification the estimated shapes of the EKCs and the turning points for three illustrative scaling factors: *a*—again labelled ‘Act’, ‘Min’, and ‘Max’ shown on the income axis. Again, this demonstrates that the estimated EKC turning points for both countries are just scaled by that used to scale the independent variable in functional form Eq. (2b). Thus, the shapes of the EKCs for each country are identical, and the turning points are at the same level of emissions, with the income just scaled accordingly. So, for the cubic case, both $\hat{\beta_1}$ and $\hat{\beta_2}$ have *no* impact on the actual shape of the estimated EKC and where the important turning point is—whether $\hat{\beta_1}$ and $\hat{\beta_2}$ are positive or negative and/or statistically significant/insignificant. Therefore, decisions by researchers on the acceptance or otherwise the existence and shape of an estimated EKC should *not* be based on $\hat{\beta_1}$and/or $\hat{\beta_2}$. Furthermore, it is interesting to note that, for this specification, the second turning point for Denmark does not fall within the sample range (unlike Sweden), which suggests that despite $\hat{\beta_3}$ being positive and significant, the cubic specification is not appropriate for Denmark (although it might be for Sweden).

**Fig. 6:**
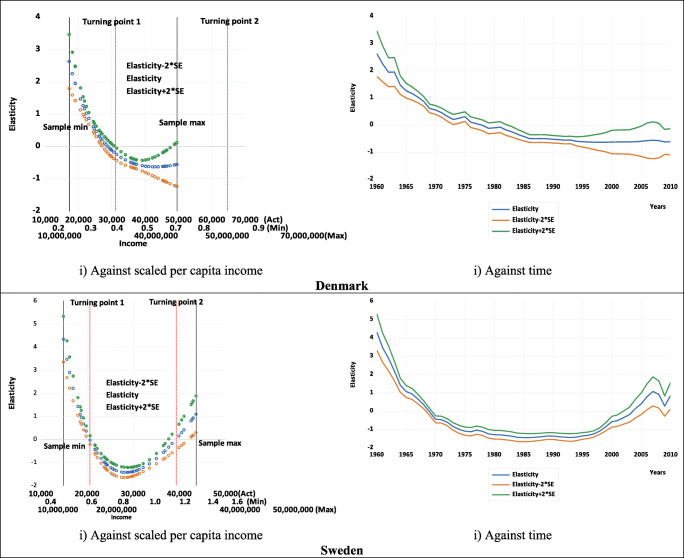
Estimated pointwise elasticities with 95% confidence intervals. (Note: For Denmark, Act, *a* =1; Min, *a* =0.000017; Max, *a* =1000; for Sweden, Act, *a* =1, Min, *a* =0.000035; Max, *a* =1000. These represent the examples of the scaling factors shown on the Denmark and Sweden income axes, respectively.)

Figure 6 displays the estimated pointwise elasticities for Denmark and Sweden against income and time using the different scaling factors. Like Fig. 3 for the quadratic case, Fig. 6 clearly shows that for the cubic case, the elasticity estimates are not unit dependent (both when plotted against income and time). For both Denmark and Sweden, the estimated pointwise elasticities are positive and significant at low levels of income but gradually fall, becoming insignificant the closer to the estimated first turning point, where the elasticity would be zero. Thereafter, as income increases, the estimated elasticities continue to fall, gradually becoming more negative and significant. However, Denmark then follows a different path to that of Sweden. For Denmark, the estimated elasticities flatten and do not reach the second turning point, given it is outside of the data range, whereas for Sweden, the estimated elasticities start to rise again, becoming around zero at the estimated second turning point and then continue to rise. As for the charts against time in the second column of Fig. 6, these show slightly different patterns but still reflect the situation in the first column. Again, it is interesting to focus on the illustrative estimates for Denmark presented in Fig. 6, since the plots against both income and time show that the cubic or N-shaped specification is not appropriate. For there to be an N-shaped pattern, the elasticity should have at least some positive values after the second turning point when plotted against income (and close to the end of the sample when plotted against time). However, this is clearly not the case for Denmark in Fig. 6, which is in line with the finding of the second turning point to be outside of the sample range for Denmark. Nonetheless, this is the case for Sweden in Fig. 6, which would be expected given both estimated turning points are within the data sample range, and Fig. 6, therefore, suggests that an estimated N-shaped EKC might be appropriate for Sweden.

### Empirical illustration of the quartic logarithmic EKC

**Fig. 7:**
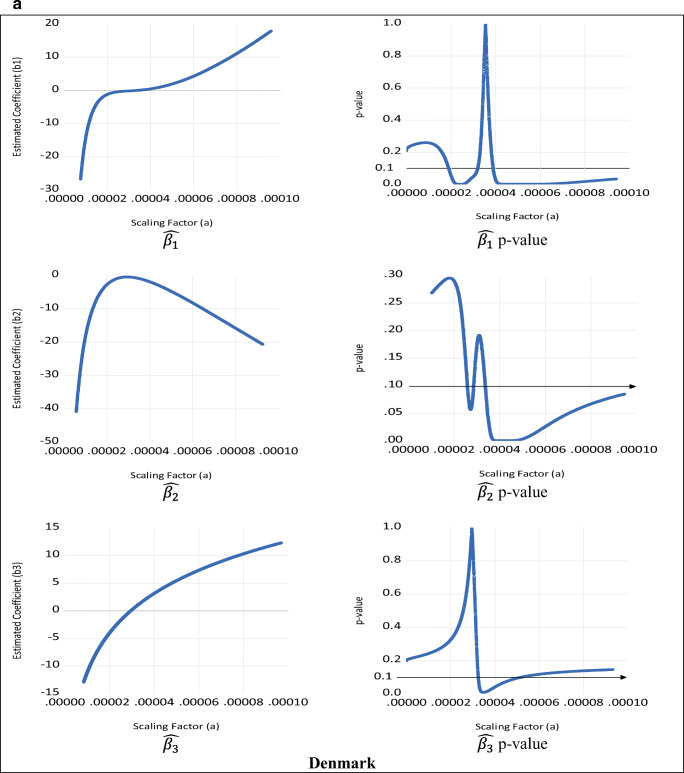

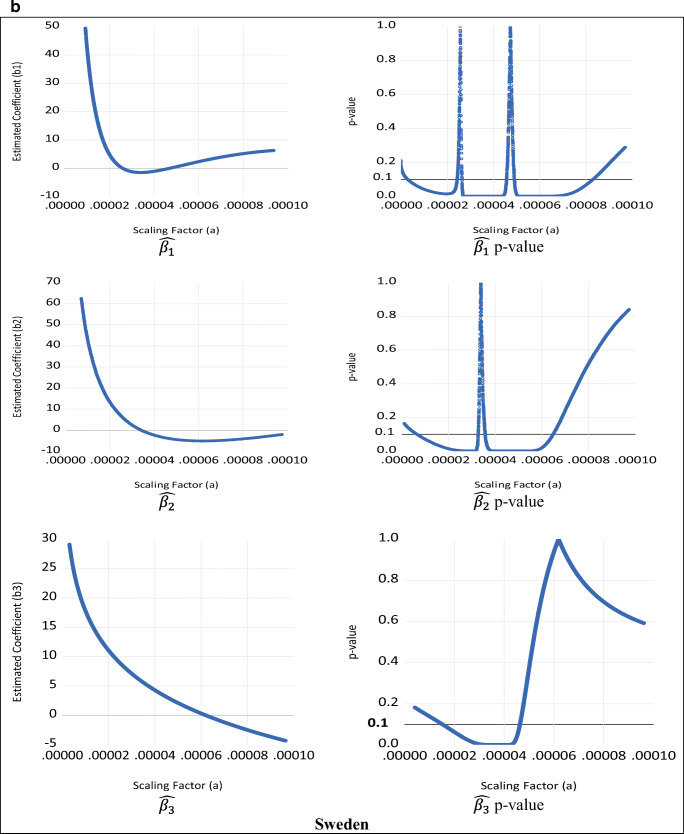
Various estimates for $\hat{\beta_1}$, $\hat{\beta_2}$, $\hat{\beta_3}$, and associated significance levels for quartic EKC—**a** Denmark, **b** Sweden

The results for the empirical estimation for the quartic case are presented in Table 3 and illustrated in Fig. 7a and 7b for a range of illustrative scaling factors. These results show that the quartic specification results are consistent with the quadratic and cubic cases. Although the size, sign, and significance of the estimated coefficients of the leading term ($\hat{\beta_4}$) are unit independent, this is not the case for $\hat{\beta_1}$, $\hat{\beta_2}$, and $\hat{\beta_3}$, since Table 3 and Fig. 7a and 7b clearly show that their sign, size, and significance are generally unit dependent. Moreover, there is a range of scaling factors (illustrated by the horizontal 10% significance line in the second column of Fig. 7a and 7b) for which the coefficients $\hat{\beta_1}$, $\hat{\beta_2}$, and $\hat{\beta_3}$ become statistically insignificant. Furthermore, like the quadratic and cubic cases, the elasticities and their *t*-values (and thus their statistical significance) are invariant to rescaling. It is interesting to note that, for the quartic case, the leading term ($\hat{\beta_4}$) and the summary elasticity ($\hat{\eta}$) are insignificant for both Denmark and Sweden. This suggests that the quartic specification is not the appropriate representation of the EKC for both countries.

**Table 3:** Quartic specification estimation results

**Denmark**							
Scaling factor (*a*)	0.00003	0.00004	0.0000483	0.00009	1 (original data)	10	100
$\hat{\beta_1}$	−0.2568^**^	0.3390^**^	1.4673^***^	15.0919^**^	11762.7366	21399.8491	35237.5345
*t*-value	(−2.0577)	(2.1484)	(8.7833)	(2.2716)	(1.3781)	(1.3682)	(1.3615)
$\hat{\beta_2}$	−0.5644	−1.9360^***^	−4.2326^***^	−19.6682^*^	−1691.6130	−2521.2378	−3515.8986
*t*-value	(−1.4283)	(−5.7221)	(−3.5257)	(−1.7923)	(−1.3599)	(−1.3536)	(−1.3494)
$\hat{\beta_3}$	0.0968	3.0818^**^	5.0382^*^	11.4959	108.1548	132.0462	155.9376
*t*-value	(0.0931)	(2.1308)	(1.7544)	(1.4912)	(1.3427)	(1.3398)	(1.3378)
$\hat{\beta_4}$	−2.5940	−2.5940	−2.5940	−2.5940	−2.5940	−2.5940	−2.5940
*t*-value	(−1.3267)	(−1.3267)	(−1.3267)	(−1.3267)	(−1.3267)	(−1.3267)	(−1.3267)
$\hat{\eta}$	−0.2726	−0.2726	−0.2726	−0.2726	−0.2726	−0.2726	−0.2726
*t*-value	(−0.0299)	(−0.0299)	(−0.0299)	(−0.0299)	(−0.0299)	(−0.0299)	(−0.0299)
**Sweden**							
Scaling factor (*a*)	0.00003	0.00004	0.0000483	0.00007	1 (original data)	10	100
$\hat{\beta_1}$	−1.2502^***^	−1.1679^***^	0.1306	3.8387^***^	−8813.4848	−16778.0579	−28482.9102
*t*-value	(−7.6954)	(−9.5781)	(0.7235)	(2.9922)	(−0.9615)	(−0.9912)	(−1.0116)
$\hat{\beta_2}$	2.5221^***^	−2.4026^***^	−4.3097^***^	−5.0085	1375.3762	2109.5873	2999.7589
*t*-value	(4.5660)	(−4.1894)	(−9.5357)	(−1.2014)	(1.0153)	(1.0348)	(1.0482)
$\hat{\beta_3}$	7.1166^***^	4.2958^***^	2.4470	−1.1914	−94.9991	−117.5767	−140.1543
*t*-value	(3.0627)	(6.5917)	(1.2162)	(−0.2289)	(−1.0682)	(−1.0777)	(−1.0843)
$\hat{\beta_4}$	2.4513	2.4513	2.4513	2.4513	2.4513	2.4513	2.4513
*t*-value	(1.1199)	(1.1199)	(1.1199)	(1.1199)	(1.1199)	(1.1199)	(1.1199)
$\hat{\eta}$	−1.5508	−1.5508	−1.5508	−1.5508	−1.5508	−1.5508	−1.5508
*t*-value	(−0.2201)	(−0.2201)	(−0.2201)	(−0.2201)	(−0.2201)	(−0.2201)	(−0.2201)

Source: Data from Jaforullah and King (2017)

Notes: The values of the scaling factors are chosen to show the existence of coefficients with the opposite signs and with significant and insignificant values (according to *t*-values). The estimated elasticities $\hat{\Big(\eta}\Big)$ are calculated at mean values as discussed in Gujarati and Porter (2009), inter alia

**Fig. 8:**
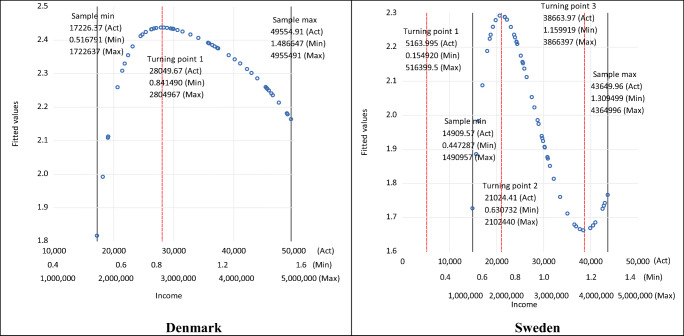
Estimated turning points with scaled per capita income. (Notes: Act, *a* =1; Min, *a* =0.00003; Max, *a* =100. These represent the examples of the scaling factors shown on the income axes. For Denmark, only one turning point is real, and other two turning points (roots) are complex.)

Figure 8 illustrates the estimated shapes of the EKCs and the turning points for three illustrative scaling factors for the quartic case—once again labelled as ‘Act’, ‘Min’, and ‘Max’ shown on the income axis. Like before, this shows that the estimated EKC turning points are identical, and the turning points are at the same level of emissions but with income just scaled accordingly. So in this case, $\hat{\beta_1}$, $\hat{\beta_2}$, and $\hat{\beta_3}$ have *no* impact on the actual shape of the estimated EKC and where the important turning points are. Whether $\hat{\beta_1}$, $\hat{\beta_2}$, and/or $\hat{\beta_3}$ are positive/negative and/or statistically significant/insignificant has no impact on the estimated EKC whatsoever. Therefore, decisions by researchers on the acceptance or otherwise the existence and shape of an estimated quartic EKC should *not* be based on $\hat{\beta_1}$, $\hat{\beta_2}$, and/or $\hat{\beta_3}$.

In terms of the actual shapes, it is interesting to note that only one turning point is established for Denmark since the other two have complex roots. This is consistent with the cubic case where the second turning point was outside the data sample range—all pointing to the EKC for Denmark being a simple quadratic function—whereas for Sweden, all three turning points are determined, but the first one is not within the data sample range. This coupled with the insignificant $\hat{\beta_4}$ suggests that for Sweden also, the quartic specification is not the appropriate one, but the cubic specification is.

**Fig. 9:**
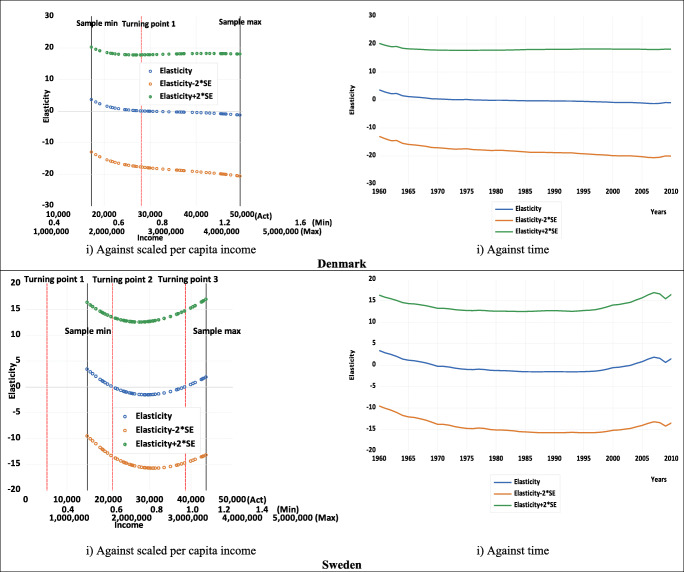
Estimated pointwise elasticities with 95% confidence intervals. (Notes: Act, *a* =1; Min, *a* =0.00003; Max, *a* =100. These represent the examples of the scaling factors shown on the income axes. For Denmark, only one turning point is real, and other two turning points (roots) are complex.)

Finally, for completeness, Fig. 9 displays the estimated pointwise elasticities for both countries against income and time using the different scaling factors and again shows that the elasticity estimates are not unit dependent. Since the pointwise elasticity is insignificant for both country cases, it confirms what has been suggested above in terms of the insignificance of the leading term and the turning points: the quartic specification is not the appropriate representation of the EKC relationship for either country.

### Summary

The empirical illustrations presented in this section clearly highlight the potential problem when estimating non-linear logarithmic EKCs. We have shown that the signs, sizes, *t*-values, and statistical significances of the estimated coefficients of the variables other than the leading term (the variables with the highest power) *are unit dependent*, whereas the sign, size, *t*-value, and statistical significance of the estimated coefficient of the leading term (the variable with the highest power of the polynomial functional form) are *unit of measurement invariant*. Furthermore, we have also shown that the estimated elasticities and their *t*-values (and consequentially their statistical significances) are also invariant to any rescaling (they are unit of measurement invariant), while the turning points, although rescaled by the same rescaling factor, are effectively the same.

We have also highlighted the factors that should be considered when choosing the preferred specification for an estimated logarithmic EKC. The focus should clearly be on the sign and size of the leading term, the elasticity estimates, and where the turning point(s) are relative to the sample data range. Researchers should not just choose a certain polynomial and estimate it blindly without a detailed scrutiny of all the factors discussed above. The next section summarises and discusses the consequences of these issues and details a suggested way forward for future researchers when estimating logarithmic EKCs.

## Summary and Conclusion

Understanding the relationship between environmental pollution and its different drivers is a major issue in the environmental economics literature, with much research focussed on attempting to find the existence or otherwise of EKCs. The use of an appropriate functional form for the EKC and the proper interpretation of findings are at the heart of this research. As Jaforullah and King (2017) and Mikayilov et al. (2018), inter alia, discuss, using inappropriate functional forms can result in a relationship that does not properly demonstrate the response of pollution to its drivers. Moreover, when using different polynomials with the variables in logarithmic form (as shown here with the quadratic, cubic, and quartic specifications), it is important to understand the effect of measuring the variables in different units of measurement and how to choose an appropriate preferred estimated EKC specification. In this regard, this paper investigates the effect on the *estimated coefficients*, their *t*-values and *statistical significance*, the *turning points* of the function, and the *estimated elasticities*. It shows that:

I. The signs, sizes, *t*-values, and statistical significance of the estimated coefficient of the non-leading terms are unit dependent, rendering the estimates arbitrary. *Thus, they should not contribute to any decision about the conclusion of the existence of an EKC nor its shape*.
II. The sign, size, *t*-value, and statistical significance of the estimated coefficient of the leading term are unit independent. *Thus, this should contribute to the decision about the conclusion of the existence of an EKC and its shape.*
III. Rescaling the independent income variable causes the turning point to also be rescaled, but its location with respect to the minimum and maximum value of emissions stays unchanged. *Thus*, *this should contribute to the decision about the conclusion of the existence of an EKC and its shape*.
IV. The signs, sizes, *t*-values, and significance of estimated elasticities, like the estimate of the coefficient of the leading term, are also unit independent. *Thus, these should contribute to the decision about the conclusion of the existence of an EKC and its shape.*

This clearly demonstrates that the practice followed in many previously published environmental economics papers, whereby the estimates of the non-leading terms are used in the decision to choose the preferred specification of an estimated logarithmic EKC, is incorrect and should **not** be followed since it could result in misleading conclusions. Instead, we suggest the following strategy should be adopted by researchers for determining the existence and shape of an estimated logarithmic EKC.

- Given the potential problems with the logarithmic specification, researchers should initially investigate whether it might be appropriate to estimate the EKC in levels, like in Eq. (1), or in logarithms, like in Eq. (2). For this, following Moosa (2017), we suggest that non-nested tests (such as those detailed in Pesaran and Pesaran 2009) are conducted to try to determine which would be the most appropriate, since some of the issues outlined in this paper might be avoided if it is clear that a level version of the EKC like those in Eq. (1) is preferred. If, however, the logarithmic version of the EKC, like those in Eq. (2), is preferred (or, as sometimes happens with non-nested tests, a clear distinction between the specifications is not possible), then the criteria (set out in b) below should be followed.
- Estimate a polynomial logarithmic EKC like those presented in Eq. (2), with the initial order of the polynomial chosen by the researcher. However, given the growing interest in the literature in attempting to estimate higher-order specifications, this choice should be ‘reasonably high’ to be able to test down in the spirit of the ‘general-to-specific’ methodology, based on the following criteria.
  i. Check the statistical significance of the leading term; i.e. check whether *β*_4_ is significant if estimating a quartic specification as in Eq. (2c), or whether *β*_3_ is significant if estimating a cubic specification as in Eq. (2b), or whether *β*_2_ is significant if estimating a quadratic specification as in Eq. (2a).
    *If the leading term is not statistically significant, try re-estimating with a lower-order polynomial.*
    *If the leading term is statistically significant, go to ii.*
  ii. Check the sign of the leading term to ensure that the estimates conform with the a priori expectations about the shape of the EKC; i.e. for a quartic specification as in Eq. (2c), if *β*_4_ < 0, it suggests an M-shaped relationship; for a cubic specification as in Eq. (2b), if *β*_3_ > 0, it suggests an N-shaped relationship; and for a quadratic specification as in Eq. (2a), if *β*_2_ < 0, it suggests an inverted U-shaped relationship.^20^ Furthermore, if the estimates do not conform with a priori expectations, then a judgement is needed as to whether the estimated shape suggested is acceptable or not.
    *If it is decided that the estimated shape is not acceptable, then alternative specifications would need to be explored.*^21^
    *If it is decided that the estimated shape is acceptable, then go to iii to further test the acceptability of the estimated model.*
  iii. Check that all the acceptable estimated turning points for the specification estimated, be it quartic, cubic, or quadratic, are within a reasonable range—i.e. greater than the sample minimum value and smaller than the sample maximum value.
    *If the turning points are not within the reasonable range, then alternative specifications would need to be explored.* For example, if for an estimated M-shaped quartic EKC the income level for the highest turning point is found to be higher than the sample maximum income value, or the income level for the highest turning point is found to be higher than the sample maximum income value for an estimated N-shaped cubic EKC, then it would suggest that lower polynomials should be explored. Ultimately, if the income level for the highest turning point is found to be higher than the sample maximum income value for an estimated inverted U-shaped quadratic EKC, in all probability, it would suggest that the PIR is monotonically increasing. In short, we suggest a ‘general-to-specific’ type approach until a statistically acceptable model consistent with a priori expectations is obtained.^22^
    *If, however, the turning points are within the reasonable range, then go to iv to further test the acceptability of the estimated model.*
  iv. Check that the estimated summary elasticity is significant and that the pointwise elasticities follow the pattern in terms of the sign, size, and significance, as discussed and shown in Fig. 3, Fig. 6, and Fig. 9 for the appropriate EKC polynomial being estimated. In other words, for any estimated EKC, be it inverted U-shaped, N-shaped, or M-shaped, the estimated pairwise elasticities should be positive and significant for the initial upward sloping part of the estimated curve, but they approach zero and become insignificant at the first turning point, thereafter becoming negative and significant on the downward sloping part. If, however, an N- or M-shaped EKC is being considered, after the first turning point, the estimated pairwise elasticities should continue to be negative and significant but approach zero and become insignificant at the second turning point, thereafter becoming positive again and significant on the next upward sloping part of the estimated curve. If, however, an M-shaped EKC is being considered, the estimated pairwise elasticities should continue to be positive and significant after the second turning point, but they approach zero and become insignificant at the third turning point, thereafter becoming negative again and significant on the next downward sloping part of the estimated curve.^23^
    *If this condition is not satisfied, then again alternative specifications would need to be explored.*^24^
    *However, if this final condition is accepted following the acceptance of all the previous conditions, then the estimated relationship could be considered as the preferred estimate for appropriate economic and policy analysis.*

This paper has explored important issues around estimating logarithmic EKCs using various orders of polynomial specifications in detail. It highlights some of the common pitfalls but also develops a modelling strategy that should ensure a consistent approach for researchers investigating logarithmic EKCs. 25 That said, this paper focuses very much on the one type of specification, albeit an extremely popular type, and when undertaking such modelling, it is vital that the correct and appropriate econometric estimation and testing techniques are employed to the highest standard and considered alongside the modelling strategy suggested above. Moreover, future research should consider how the non-linear polynomial specifications considered in this paper could be used, or compared, with econometric non-linear modelling methods as discussed in Lieb (2003), Dinda (2004), and Kijima et al. (2010), inter alia. These include the structural time series method (Harvey 1989); time-varying coefficient cointegration (Park and Hahn 1999); quantile cointegration regression (Xiao 2009); and the multiplicative indicator saturation approach (Ericsson 2012; Castle and Hendry 2019).

## Supplementary Information

ESM 1 (PDF 266 kb)

## Footnotes

1 Some alternative names have also been suggested such as the carbon Kuznets curve (CKC) by Zhang et al. (2019).

2 Note, for simplicity, the mathematical explanation in Section 3 focuses on the quadratic case, but the results can easily be generalised for the cubic and quartic cases (they are given in Appendix 2 for completeness).

3 Note that we specify the variables in per capita form like most papers that attempt to estimate EKCs, although some studies do not. See, for example, Friedl and Getzner (2003).

4 For illustrative purposes, all equations are specified in a time series context, but the issues raised in the paper apply equally to panel data estimates.

5 Several studies have included additional right-hand variables, such as energy consumption and a time trend (see, e.g. Lieb 2003; Paramati et al. 2016; Qureshi et al. 2017; Ang 2007 and many others), but these are ignored in this paper, given its focus. Nonetheless, the issues raised in this paper are not affected by the inclusion or exclusion of the added variables (provided they are not included logarithmically and multiplicatively). Hence, they are ignored here.

6 Note, for clarity, the notation used here is that *ln*^*n*^*X*_*t*_=(*lnX*_*t*_)^*n*^.

7 Note that the following specifications could also be used, and some have, but less frequently than Eqs. (1) and (2). *Semi-logarithmic (log-lin)*

$$ {\displaystyle \begin{array}{c}\mathit{\ln}{E}_t={\gamma}_0+{\gamma}_1{Y}_t+{\gamma}_2{Y}_t^2\\ {}\mathit{\ln}{E}_t={\gamma}_0+{\gamma}_1{Y}_t+{\gamma}_2{Y}_t^2+{\gamma}_3{Y}_t^3\\ {}\mathit{\ln}{E}_t={\gamma}_0+{\gamma}_1{Y}_t+{\gamma}_2{Y}_t^2+{\gamma}_3{Y}_t^3+{\gamma}_4{Y}_t^4\end{array}} $$

*Semi-logarithmic (lin-log)*

$$ {\displaystyle \begin{array}{c}{E}_t={\delta}_0+{\delta}_1\mathit{\ln}{Y}_t+{\delta}_2{\mathit{\ln}}^2{Y}_t\\ {}{E}_t={\delta}_0+{\delta}_1\mathit{\ln}{Y}_t+{\delta}_2{\mathit{\ln}}^2{Y}_t+{\delta}_3{\mathit{\ln}}^3{Y}_t\\ {}{E}_t={\delta}_0+{\delta}_1\mathit{\ln}{Y}_t+{\delta}_2{\mathit{\ln}}^2{Y}_t+{\delta}_3{\mathit{\ln}}^3{Y}_t+{\delta}_4{\mathit{\ln}}^4{Y}_t\end{array}} $$

See, for example, Lieb (2002) who estimated semi-logarithmic versions of an EKC (where *either* pollution *or* income was in logs).

8 Hunt and Lynk (1993) illustrated this empirically by estimating a translog production function.

9 Note, unlike other examples, Bimonte and Stabile (2017) find a U-shaped relationship based on the sign of the estimated coefficients of the leading term *and* the non-leading term; hence, this has the same problem as the other examples for a U-shaped relationship.

10 This point can also be explained geometrically. For the logarithmic scale, the coordinates are (*lnY*_*t*_, *lnE*_*t*_). However, the rescaling *ln*(*aY*_*t*_) =  *ln Y*_*t*_ + *lna* can be considered as a transformation (a parallel shift) of the coordinates. In other words, the points of the form (*lnY*_*t*_, *lnE*_*t*_) are transformed to (*lnY*_*t*_ + *lna*, *lnE*_*t*_), or equally the abscissa is being shifted by (*lna*) unit horizontally. The ordinate remains the same (see Figs. 2, 4, and 6) since the points of the plane shift horizontally. The roots of the polynomial, the intersection points with abscissa line, and the intersection point with ordinate axes will change. Hence, based on Vieta’s (1579) formulas, the coefficient of the second term and the intercept will change. However, the ordinate axes will not be affected as with the coefficient of the leading term.

11 Note we are not trying to pretend that this is a new finding as such, but what is new is that it is the *only* necessary condition for a quadratic (inverted U-shaped) logarithmic EKC. For example, the analytical section of Sinha et al. (2018) implicitly derives the necessary conditions for a quadratic non-logarithmic EKC like Eq. (1a) as being *α*_1_ > 0 and *α*_2_ < 0; however, we are explicitly concerned with the quadratic (inverted U-shaped) logarithmic version of the EKC where, as we show, it is only the leading term, *β*_2_, that is relevant.

12 Taking the derivative of Eq. (2a) gives $\frac{dE}{dY}=\frac{E}{Y}\left({\beta}_1+2{\beta}_2 lnY\right)$, and equating the left-hand side of this expression to zero results in $\frac{E}{Y}=0\  or\ \left({\beta}_1+2{\beta}_2 lnY\right)=0$. Solving the second equation, we end up with ${\beta}_1+2{\beta}_2 lnY=0\overset{yields}{\to } lnY=-\frac{\beta_1}{2{\beta}_2}\overset{yields}{\to }\ Y=\exp \left(-\frac{\beta_1}{2{\beta}_2}\right).$

13 Again, this point can be explained geometrically. For the logarithmic scale, the coordinates are (*lnY*_*t*_, *lnE*_*t*_). However, the rescaling *ln*(*aY*_*t*_) =  *ln Y*_*t*_ + *lna* can be considered as a transformation (a parallel shift) of the coordinates, and the points of the form (*lnY*_*t*_, *lnE*_*t*_) are transformed to (*lnY*_*t*_ + *lna*, *lnE*_*t*_), or equally the abscissa is being shifted by (*lna*) unit horizontally. The ordinate remains the same (see Fig. 2), while the abscissa of the turning point is also shifted by (*lna*) horizontally.

14 Namely,

$$ \mathit{\operatorname{var}}\left(a{x}_1+b{x}_2\right)={a}^2\ast \mathit{\operatorname{var}}\left({x}_1\right)+2 ab\ast \mathit{\operatorname{cov}}\left({x}_1,{x}_2\right)+{b}^2\ast \mathit{\operatorname{var}}\left({x}_2\right) $$

$$ \mathit{\operatorname{cov}}\left(a{x}_1+b{x}_2,c{x}_3\right)= ac\ast \mathit{\operatorname{cov}}\left({x}_1,{x}_3\right)+ bc\ast \mathit{\operatorname{cov}}\left({x}_2,{x}_3\right) $$

15 As mentioned in previous footnotes, the ordinate remains the same since the points of the plane shift horizontally. The roots of the polynomial, the intersection points with abscissa line, and the intersection point with ordinate axes will change. However, the ordinate axes will not be affected, as with the coefficient of the leading term. Since the position of the function with respect to the x-axes does not change directionally, the position of the tangent line at any point of the function is not affected by this parallel shift. Hence, elasticity, which is the geometric interpretation of a derivative at a certain point of a function (in logarithmic scale), remains invariant.

16 Note, $-\frac{\beta_1}{2{\beta}_2}$ is derived from equating the elasticity function, Eq. (11a), to zero and is identical to finding the turning point, Eq. (10a).

17 The data are available online at 10.1016/j.eneco.2017.01.025 (Jaforullah and King 2017) and are used given they have been used for a previous EKC estimation. However, they are purely used as an illustration of the points highlighted in this paper.

18 Note, for completeness, we also used the autoregressive distributed lag (ARDL) bounds testing approach (Pesaran and Shin 1999; Pesaran et al. 2001) used by Jaforullah and King (2017) as a check and found the same results as in their paper.

19 Furthermore, it is worth highlighting that due to the fact that the sign, size, and significance of *β*_1_ change according to the rescaling, it cannot be used for the interpretation of the impact of economic growth on environmental degradation, as in Al-Mulali et al. (2016), Shahbaz et al. (2018), and Sharif et al. (2019). Instead, the estimated elasticity should be used for this purpose.

20 Stages i. and ii. are analogous to checking the *necessary* conditions discussed in Section 3 and Appendix 2.

21 This includes where for a quartic specification *β*_4_ > 0 and statistically significant, for the cubic specification *β*_3_ < 0 and statistically significant, and for the quadratic specification *β*_2_ > 0 and statistically significant suggesting an inverted M-shaped/W-shaped, inverted N-shaped, or U-shaped EKC, respectively. Although such specifications have been acceptable in some parts of the literature, we argue that this is unlikely to be a true representation of an economy’s initial development stage, and hence should not be acceptable, and alternative specifications should be explored.

22 However, it should not be forgotten that standard econometric testing should be applied when searching for the preferred EKC specification, such as in the case of small sample problems (Juselius 2006), discussed in more detail in Appendix 1. What we are suggesting here is *additional* to undertaking sound state-of-the-art econometric estimation and testing.

23 Stages ii and iv are analogous to checking the *sufficient* conditions discussed in Section 3 and Appendix 2.

24 It is worth noting that in some cases, a quantile regression approach has been used to estimate a PIR by identifying different regimes for the different upward and downward sloping parts of the EKC (see Liddle and Messinis 2016 and Apergis 2016, inter alia). However, non-linear techniques like these do not have a formal modelling strategy like we outline but require an assumption about the different number of regimes, which potentially might result in arbitrary results.

25 Although the focus here is on the Kuznets curve relationship applied to environmental degradation, the principles developed and suggested here equally apply to any attempt to estimate econometrically a logarithmic Kuznets curve relationship. These include energy Kuznets curves such as Luzzati and Orsini (2009) and even suicidal Kuznets curves such as Antonakakis and Collins (2018). A similar issue is also one of the most discussed topics in the so-called non-linear production function approach (see, Meyer and Kadiyala 1974; Corbo and Meller 1979; Boisvert 1982; Heathfield and Wibe 1987, inter alia).

## References

[CR1] Al-Mulali U, Ozturk I, Solarin SA (2016). Investigating the environmental Kuznets curve hypothesis in seven regions: the role of renewable energy. Ecol Indic.

[CR2] Alshehry AS, Belloumi M (2016). Energy consumption, carbon dioxide emissions and economic growth: the case of Saudi Arabia. Renew Sust Energ Rev.

[CR3] Ang JB (2007). CO_2_ emissions, energy consumption, and output in France. Energy Policy.

[CR4] Antonakakis N, Collins A (2018). A suicidal Kuznets curve?. Econ Lett.

[CR5] Apergis N (2016). Environmental Kuznets curves: new evidence on both panel and country-level CO_2_ emissions. Energy Econ.

[CR6] Arshad Z, Robaina M, Shahbaz M, Veloso AB (2020). 2020. The effects of deforestation and urbanization on sustainable growth in Asian countries. Environ Sci Pollut Res.

[CR7] Atici C (2008). Carbon emissions in Central and Eastern Europe: environmental Kuznets curve and implications for sustainable development. Sustain Dev.

[CR8] Auci S, Becchetti L (2006). The instability of the adjusted and unadjusted environmental Kuznets curves. Ecol Econ.

[CR9] Baek J (2015). Environmental Kuznets curve for CO_2_ emissions: the case of Arctic countries. Energy Econ.

[CR10] Bagliani M, Bravo G, Dalmazzone S (2008). A consumption-based approach to environmental Kuznets curves using the ecological footprint indicator. Ecol Econ.

[CR11] Balaguer J, Cantavella M (2018). The role of education in the environmental Kuznets curve. Evidence from Australian data. Energy Econ.

[CR12] Beckerman W (1992). Economic growth and the environment: whose growth? Whose environment?. World Dev.

[CR13] Bimonte S, Stabile A (2017). Land consumption and income in Italy: a case of inverted EKC. Ecol Econ.

[CR14] Boisvert RN (1982) “The translog production function: its properties, its several interpretations and estimation problems,” Research Bulletins 182035, Cornell University, Department of Applied Economics and Management

[CR15] Castle JL, Hendry DF (2019) Modelling our changing world. Palgrave Texts in Econometrics. Gewerbestrasse 11, 6330 Cham, Switzerland. 10.1007/978-3-030-21432-6. Accessed 11 June 2020

[CR16] Choumert J, Motel PC, Dakpo HK (2013). Is the environmental Kuznets curve for deforestation a threatened theory? A meta-analysis of the literature. Ecol Econ.

[CR17] Corbo V, Meller P (1979). The translog production function: some evidence from establishment data. J Econ.

[CR18] Destek MA, Shahbaz M, Okumus I, Shawkat HA, Sinha (2020). The relationship between economic growth and carbon emissions in G-7 countries: evidence from time-varying parameters with a long history. Environ Sci Pollut Res.

[CR19] Dinda S (2004). Environmental Kuznets curve hypothesis: a survey. Ecol Econ.

[CR20] Dinda S (2005). A theoretical basis for the environmental Kuznets curve. Ecol Econ.

[CR21] Ericsson NR (2012) Detecting crises, jumps, and changes in regime; working paper. Federal Reserve Board of Governors, Washington, DC, USA

[CR22] Fosten J, Morley B, Taylor T (2012). Dynamic misspecification in the environmental Kuznets curve: evidence from CO_2_ and SO_2_ emissions in the United Kingdom. Ecol Econ.

[CR23] Friedl B, Getzner M (2003). Determinants of CO_2_ emissions in a small open economy. Ecol Econ.

[CR24] Fürstenberger G, Wagner M (2007). Exploring the environmental Kuznets hypothesis: theoretical and econometric problems. Ecol Econ.

[CR25] Galeotti M, Lanza A, Pauli F (2006). Reassessing the environmental Kuznets curve for CO_2_ emissions: a robustness exercise. Ecol Econ.

[CR26] Gawande K, Berrens RP, Bohara AK (2001). A consumption-based theory of environmental Kuznets curve. Ecol Econ.

[CR27] Gormus S, Aydin M (2020). Revisiting the environmental Kuznets curve hypothesis using innovation: new evidence from the top 10 innovative economies. Environ Sci Pollut Res.

[CR28] Grossman GM, Goldin I, Winters LA (1995). Pollution and growth: what do we know. The economics of Sustainable Development.

[CR29] Grossman GM, Krueger AB (1991). Environmental impacts of a North American Free Trade Agreement. National Bureau of Economic Research Working Paper 3914.

[CR30] Grossman GM, Krueger AB (1995). Economic growth and the environment. Q J Econ.

[CR31] Gujarati DN, Porter DC (2009). Basic econometrics.

[CR32] Harbaugh W, Levinson A, Wilson DM (2002). Re-examining the empirical evidence for an environmental Kuznets curve. Rev Econ Stat.

[CR33] Harvey AC (1989) Forecasting, structural time series models and the Kalman filter. Cambridge University Press, Cambridge, UK

[CR34] Hasanov FJ, Mikayilov JI, Mukhtarov S, Suleymanov E (2019). Does CO 2 emissions–economic growth relationship reveal EKC in developing countries? Evidence from Kazakhstan. Environ Sci Pollut Res.

[CR35] Heathfield DF, Wibe S (1987) The translog function. In: An Introduction to Cost and Production Functions. Palgrave, London

[CR36] Hong SH, Wagner M (2008) Nonlinear cointegration analysis and the environmental Kuznets curve. Economics Series 224, Institute for Advanced Studies. http://hdl.handle.net/10419/72720

[CR37] Hunt LC, Lynk EL (1993). The interpretation of coefficients in multiplicative-logarithmic functions. Appl Econ.

[CR38] Jaforullah M, King A (2017). The econometric consequences of an energy consumption variable in a model of CO_2_ emissions. Energy Econ.

[CR39] Jebli MB, Youssef SB (2015). The environmental Kuznets curve, economic growth, renewable and non-renewable energy, and trade in Tunisia. Renew Sust Energ Rev.

[CR40] Jiang L, He S, Zhong Z, Zhou H, He L (2019). Revisiting environmental Kuznets curve for carbon dioxide emissions: the role of trade. Struct Chang Econ Dyn.

[CR41] Juselius K (2006). The cointegrated VAR model: methodology and applications.

[CR42] Kasman A, Duman YS (2015). CO2 emissions, economic growth, energy consumption, trade and urbanization in new EU member and candidate countries: a panel data analysis. Econ Model.

[CR43] Khanna N, Plassmann F (2004). The demand for environmental Kuznets curve hypothesis. Ecol Econ.

[CR44] Kijima M, Nishide K, Ohyama A (2010). Economic models for the environmental Kuznets curve: a survey J. Econ Dynam Contr.

[CR45] Kuznets S (1955). Economic growth and income inequality. Am Econ Rev.

[CR46] Li W, Yang G, Li X, Sun T, Wang J (2019). Cluster analysis of the relationship between carbon dioxide emissions and economic growth. J Clean Prod.

[CR47] Liddle B, Messinis G (2016). Revisiting carbon Kuznets curves with endogenous breaks modeling: evidence of decoupling and saturation (but few inverted-Us) for individual OECD countries. Empir Econ.

[CR48] Lieb CM (2002). The environmental Kuznets curve and satiation: a simple static model. Environ Dev Econ.

[CR49] Lieb CM (2003) The environmental Kuznets curve: a survey of the empirical evidence and of possible causes. University of Heidelberg. Discussion Paper No. 391

[CR50] Maslow AH (1943). A theory of human motivation. Psychol Rev.

[CR51] Meyer RA, Kadiyala KR (1974). Linear and nonlinear estimation of production functions. South Econ J.

[CR52] Mikayilov JI, Hasanov FJ, Galeotti M (2018). Decoupling of CO_2_ emissions and GDP: a time-varying cointegration approach. Ecol Indic.

[CR53] Mills Busa JH (2013). Dynamite in the EKC tunnel? Inconsistencies in resource stock analysis under the environmental Kuznets curve hypothesis. Ecol Econ.

[CR54] Moosa IA (2017). The econometrics of the environmental Kuznets curve: an illustration using Australian CO2 emissions. Appl Econ.

[CR55] Onafowora OA, Owoye O (2014). Bounds testing approach to analysis of the environment Kuznets curve. Energy Econ.

[CR56] Panayotou T (1993) Empirical test and policy analysis of environmental degradation at different stages of economic development. World Employment Research Programme, Working Paper, International Labour Office, Geneva

[CR57] Paramati SR, Alam MS, Chen CF (2016). The effects of tourism on economic growth and CO_2_ emissions a comparison between developed and developing economies. J Travel Res.

[CR58] Park JY, Hahn SB (1999). Cointegrating regressions with time varying coefficients. Econ Theory.

[CR59] Pesaran B, Pesaran MH (2009). Time series econometrics: using Microfit 5.0..

[CR60] Pesaran M, Shin Y, Strom S (1999). An autoregressive distributed lag modeling approach to cointegration analysis. Econometrics and Economic Theory in the 20th Century: The Ragnar Frisch Centennial Symposium.

[CR61] Pesaran MH, Shin Y, Smith RJ (2001). Bounds testing approaches to the analysis of level relationships. J Appl Econ.

[CR62] Qureshi MI, Hassan MA, Hishan SS, Rasli AM, Zaman K (2017). Dynamic linkages between sustainable tourism, energy, health and wealth: Evidence from top 80 international tourist destination cities in 37 countries. J Clean Prod.

[CR63] Roca J, Padilla E, Farre M, Galetto V (2001). Economic growth and atmospheric pollution in Spain: discussing the environmental Kuznets curve hypothesis. Ecol Econ.

[CR64] Romero-Avila D (2008). Questioning the empirical basis of the environmental Kuznets curve for CO_2_: new evidence from a panel stationarity test robust to multiple breaks and cross-dependence. Ecol Econ.

[CR65] Rothman DS (1998). Environmental Kuznets curves – real progress or passing the buck? A case for consumption-based approaches. Ecol Econ.

[CR66] Schindler DW (1996). The environment, carrying capacity and economic growth. Ecol Appl.

[CR67] Selden TM, Song D (1994). Environmental quality and development: is there a Kuznets curve for air pollution emissions. J Environ Econ Manag.

[CR68] Shafik N, Bandyopadhyay S (1992). Economic growth and environmental quality: time series and cross-country evidence.

[CR69] Shahbaz M, Khraief N, Uddin GS, Ozturk I (2014). Environmental Kuznets curve in an open economy: a bounds testing and causality analysis for Tunisia. Renew Sust Energ Rev.

[CR70] Shahbaz M, Nasir AN, Roubaud D (2018). Environmental degradation in France: the effects of FDI, financial development, and energy innovations. Energy Econ.

[CR71] Sharif A, Raza SA, Ozturk I, Afshan S (2019). The dynamic relationship of renewable and non-renewable energy consumption with carbon emission: a global study with the application of heterogeneous panel estimations. Renew Energy.

[CR72] Sinha A, Shahbaz M, Balsalobre D (2018) N-shaped environmental Kuznets curve: a note on validation and falsification. MPRA Paper No. 99313. https://mpra.ub.uni-muenchen.de/99313/1-13. Acessed 24 Mar 2021

[CR73] Sorge L, Neumann A (2020). Beyond the inverted U-shape: challenging the long-term relationship of the environmental Kuznets curve hypothesis. Econ Energy Environ Policy.

[CR74] Stern DI (2004). The rise and fall of the environmental Kuznets curve. World Dev.

[CR75] Stern DI, Common MS, Barbier EB (1996). Economic growth and environmental degradation: the environmental Kuznets curve and sustainable development. World Dev.

[CR76] Tang CF, Tan BW (2015). The impact of energy consumption, income and foreign direct investment on carbon dioxide emissions in Vietnam. Energy.

[CR77] Terrell TD (2020). Carbon flux and N- and M-shaped environmental Kuznets curves: evidence from international land use change. J Environ Econ Pol.

[CR78] Uchiyama K (2016) Environmental kuznets curve hypothesis. in: environmental kuznets curve hypothesis and carbon dioxide emissions. Springer Briefs in Economics. Springer, Tokyo. https://link.springer.com/chapter/10.1007/978-4-431-55921-4_2

[CR79] Vieta F (1579) Opera mathematica. Reprinted 1646 Leiden, Netherlands. https://www.amazon.de/exec/obidos/ASIN/B0000BTZBF/ref=nosim/mathworld02-21

[CR80] Wagner M (2008). The carbon Kuznets curve: a cloudy picture emitted by bad econometrics?. Resour Energy Econ.

[CR81] Wagner M (2012). The Phillips unit root tests for polynomials of integrated processes. Econ Lett.

[CR82] Wagner M (2015). The environmental Kuznets curve, cointegration and nonlinearity. J Appl Econ.

[CR83] Xiao Z (2009) Quantile cointegrating regression. J Econometrics 150(2):248–260

[CR84] Yang H, He J, Chen S (2015). The fragility of the environmental Kuznets curve: revisiting the hypothesis with Chinese data via an “extreme bound analysis”. Ecol Econ.

[CR85] Yang G, Sun T, Wang J, Li X (2015). Modeling the nexus between carbon dioxide emissions and economic growth. Energy Policy.

[CR86] Zhang Y, Chen X, Wu Y, Shuai C, Shen L, Ye G (2019). Peaks of transportation CO_2_ emissions of 119 countries for sustainable development: results from carbon Kuznets curve. Sustain Dev.

