# Supplementary material for: Estimating different order polynomial logarithmic environmental Kuznets curves
Source: Environ Sci Pollut Res Int. 2021 Apr 1;28(31):41965–87. doi: 10.1007/s11356-021-13463-y (PMC8355002; doi:10.1007/s11356-021-13463-y)
Supplement: Supplementary file 1 — (PDF 266 kb) [file 11356_2021_13463_MOESM1_ESM.pdf]

ESTIMATING DIFFERENT ORDER POLYNOMIAL LOGARITHMIC ENVIRONMENTAL KUZNETS CURVES

ON-LINE ANNEX

Appendix 1: Review of the EKC Literature in Environmental Economics

A1.1 Introduction

Since the early 'developing' period of the EKC phenomenon there have been several aspects debated in the environmental economics literature. These issues can be broadly grouped into 'theoretical', 'mathematical/statistical', and 'empirical', all of which are discussed below.

A1.2 Theoretical issues

The theoretical real-life representativeness of the EKC specification has been questioned from its early introduction. Beckerman (1992) suggests that high income levels are the surest way of improving environmental quality, and Panayotou (1993) describes it as "an inevitable result…" (p. 14). In contrary, Rothman (1998) mentions "… most of the researchers who examined the EKC hypothesis and others point out that this (the argument stated by the EKC hypothesis) will not 'simply' happen." (p. 178). Mills Busa (2013) mentions "continued inadequacies of the EKC hypothesis and the need to instead enlist approaches that address the specific patterns of behaviour ..." (p. 125). Choumert et al. (2013) reviewing 69 studies on the relationship between deforestation and economic development conclude that the "EKC story will not fade until theoretical alternatives are provided" (p. 26). Others questioned the limited capacity of the instruments of environmental indicators (see Schindler 1996, *inter alia*). Another concern being the use of production- versus consumption-based environmental indicators (see Rothman, 1998; Gawande et al., 2001; Bagliani, 2008, *inter alia*). Some authors also discuss different cases of the potential relationship between income and environmental

degradation, such as N-shaped, M-shaped, etc. (see Shafik and Bandyopadhyay, 1992; Grossman, 1995; Lieb, 2003, *inter alia*).

Dinda (2004) reviewing the previous literature sheds light on different theoretical aspects of the EKC phenomenon and states that “evidence for the existence of EKC is inconclusive” (p. 450) and points out the “need economic models, which properly reflect” (p. 450) the true relationship between economic activity and environment. Dinda (2005) presents a feasible theoretical justification for EKC in the framework of an endogenous growth model. Kijima et al. (2010) review different theoretical approaches that explain the EKC hypothesis and emphasize the need for developing economic models from different (theoretical and empirical) points of view.

A1.3 Empirical issues

There has been an increasingly growing body of empirical literature that has investigated the PIR and concluded different and even contradictory results. As an example, Yang et al. (2015a) tested over 140 million models using Chinese data and concluded that the environmental indicator is positively and linearly related to the income variable, rejecting the quadratic case. However, given that China is a developing economy, the results of Yang et al. (2015a) arguably support the EKC literature, suggesting that economic growth increases environmental pollution during the development stage up to some threshold level of income. In contrast, Arshad et al. (2020) find a negative impact of income on pollution for Asian developing countries. Fosten et al. (2012) using a long-data set, 1850-2002, and a developed country case (UK), and utilizing a non-linear threshold cointegration technique, concluded the existence of an EKC relationship for two environmental indicators (CO_2 and SO_2 emissions). While Liddle and Messinis (2016), Mikayilov et al., (2018), *inter alia*, conclude a positive increasing impact for many developed countries. In addition, Khanna and Plassmann (2004), using U.S. data, concluded that, for the

investigated pollutants, even the high-income households have not reached the income level after which the PIR becomes downward sloping.

Auci and Becchetti (2006), using data for 54 countries, examined the adequacy of the quadratic functional specification with and without other explanatory variables. They concluded that the model, which does not include all theoretically justified variables. might produce misleading results. Roca et al. (2001), investigating the EKC phenomenon for Spain, negate the EKC, concluding that the PIR depends on many factors, and economic growth by itself cannot solve environmental problems.

Furthermore, whether total or per capita income should be used has been discussed as an issue of concern in the literature (see for example, Selden and Song, 1994; Friedl and Getzner, 2003).

A1.4 Mathematical/statistical issues

In addition to the above-mentioned theoretical and empirical issues, there have also been concerns related to mathematical/statistical grounds on which the EKC hypothesis was based upon. They encompass the inclusion of the trend in the specification, issues related to the turning point, level variables versus logged variables, among others (see Lieb 2003, *inter alia*).

Moreover, Stern et al. (1996) and Stern (2004), *inter alia*, highlighted the importance of testing the variables utilised and the relationships for integration-cointegration properties, the lack of which was subject to spurious regression results (Engle and Granger, 1987). Romero-Avila (2008), using data for 86 countries, concluded that per capita world GDP is non-stationary, while CO_2 is found to be regime-wise trend stationary, which terminates the potential long-run relationship between the two. This, in turn, questions many EKC studies that have employed

the cointegration techniques, where the environmental indicator as a dependent variable is stationary.

Furthermore, papers by Fürstenberger and Wagner (2007), Wagner (2008), Hong and Wagner (2008), Wagner (2012) and Wagner (2015), *inter alia*, question the use of powers (such as the square of an income variable) of non-stationary variables, since the integration nature of the relationships with such variables is not yet statistically well established (see Enders, 2015, *inter alia*).

Moreover, some research attempting to investigate the PIR from different aspects, specifications, and econometric/economic points of view, address concerns such as using different functional forms, non-parametric techniques, embedding the non-linearity of the PIR etc. (see Galeotti et al., 2006; Liddle and Messinis, 2016; Apergis, 2016; Moosa, 2017; Mikayilov et al., 2018, *inter alia*). These studies again produced varying results, including a positive increasing environmental impact for many developed countries.

The reviewed literature shows that there is still some way to go before we have a clearer idea on the PIR and the theories/techniques to properly reveal it. Although there is some debate about the PIR, the response of environmental quality to economic growth, especially for the long term, is most likely to be non-linear.[1] This argument can be rationalized from both theoretical and mathematical/statistical points of view. First, from the theoretical perspective, as mentioned in the related literature (Lieb, 2003; Dinda, 2004, *inter alia*), there is a demand

[1] A similar issue is also one of the most discussed topics in the so-called non-linear production function approach (see, Meyer and Kadiyala, 1974; Corbo and Patricio, 1979; Boisvert, 1982; Heathfield and Wibe, 1987, *inter alia*).

for environmental quality, whether it is regarded as being a normal or a luxury good. At the early stage of a country or society's development, meeting the first items on the top of the demand pyramid is preferred, so controlling environmental degradation is not a top priority. Later, while the first necessities are met and society has improved its environmental awareness, a cleaner environment becomes a concern. This likely results in a change in the PIR. Therefore, from a theoretical point of view, it is rational to consider the non-linear PIR.

From the mathematical/statistical point of view, a linear relationship (constant response) is a special case of the non-linear curve (varying response), whose existence needs testing (Park and Hahn, 1999; Castle and Hendry, 2019, *inter alia*). As proposed by Lobachevskian geometry, the constant response (linear relationship) is the result of our inability to capture the big picture (non-linear relationship), which nests the constant as a shorter-period response. To put it differently, as discussed in Juselius (2006), a researcher having the same observations from x_1 to x_6 will not be able to distinguish the case in Figure A1, constant mean and variance, from the case in Figure A2 (the figures are taken from Juselius, 2006), time-varying mean and constant variance, without a longer time series.

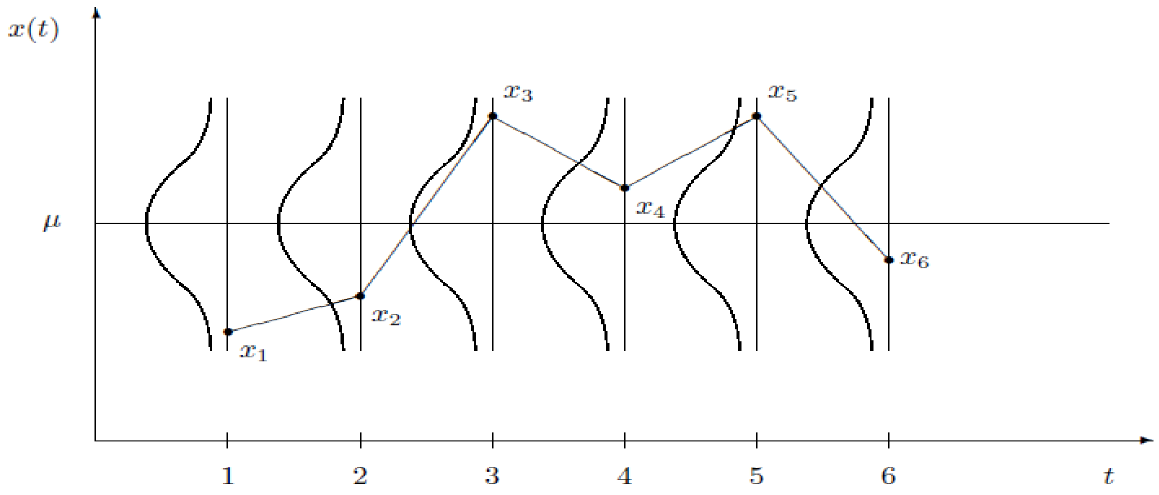

Figure A1: $E(x_t) = \mu, Var(x_t) = \sigma^2$, t=1, …, 6
(Source: Juselius, 2006)

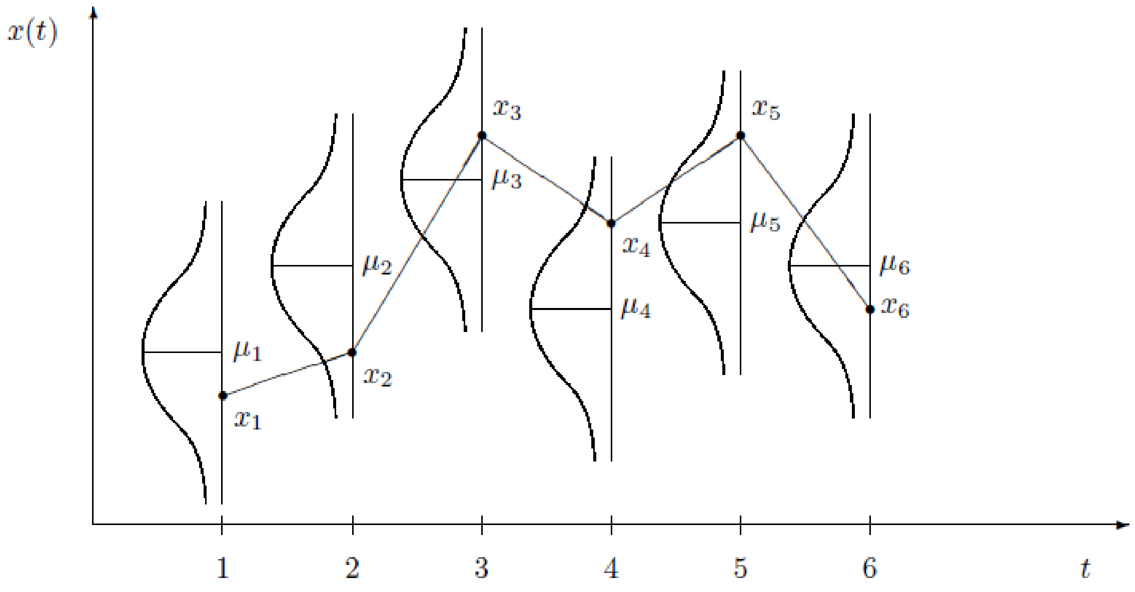

Figure A2: $E(x_t) = \mu_t, Var(x_t) = \sigma^2$, t=1, …, 6
(Source: Juselius, 2006)

Hence, the linear relationship might result from two channels. First, the country is still ‘climbing the hill’ to a better quality of life, prioritizing income and other targets over environmental quality. This can be interpreted as part of the nonlinear relationship before the peak; the PIR might not be quadratic though. Second, the investigator may be faced with a poor sample as mentioned above (example from Juselius, 2006).

Considering the above-mentioned points, it seems quite feasible to model the PIR as a non-linear relationship. In doing so, in addition to employing some other rare methods to capture non-linearities, the use of quadratic (cubic) functional specifications is still widely used to investigate the PIR (as discussed in Lieb, 2003; Dinda, 2004; Kijima et al., 2010, *inter alia*).

Considering the broader use of quadratic and other higher order polynomial functional specifications, this study uncovers, to the best of our knowledge, one of the untouched issues related to these specifications in logged variables. Namely, we investigate whether the coefficients of the higher order polynomial functional specifications are (in)variant to the rescaling of the independent variables since the existence and shape of a PIR rely on the sign, size, and statistical significance of the estimated coefficients and we therefore believe that this issue is a crucial contribution to the EKC literature.

Appendix 2: Cubic and Quartic EKC Specifications

This appendix addresses the same issue as those in the main text but focuses on the cubic and Quartic logarithmic EKC specifications, to show that the findings from the quadratic and cubic specification equally applies.

A2.1 Cubic EKC Specification

A2.1.1 Estimated parameters and statistical significance

Utilising the same scaling as in Eq. (3), Eq. (2b) becomes:

$$lnE_t = \beta_0^* + \beta_1^* lnY_t^* + \beta_2^* ln^2 Y_t^* + \beta_3^* ln^3 Y_t^* \quad \text{(A1)}$$

and substituting Eq. (3) into Eq. (A1) and rearranging gives:

$$lnE_t = [\beta_0^* + \beta_1^* lna + \beta_2^* ln^2 a + \beta_3^* ln^3 a] + [\beta_1^* + 2\beta_2^* lna + 3\beta_3^* ln^2 a] lnY_t + [\beta_2^* + 3\beta_3^* lna] ln^2 Y_t + \beta_3^* ln^3 Y_t \quad \text{(A2)}$$

Given the left-hand side of Eqs. (2b) and (A2) are the same, the two equations can be equated to give the following formulas relating the new to the original coefficients:

$$\beta_0^* = \beta_0 - \beta_1 lna + \beta_2 ln^2 a - \beta_3 ln^3 a \quad \text{(A3)}$$

$$\beta_1^* = \beta_1 - 2\beta_2 lna + 3\beta_3 ln^2 a \quad \text{(A4)}$$

$$\beta_2^* = \beta_2 - 3\beta_3 lna \quad \text{(A5)}$$

$$\beta_3^* = \beta_3 \quad \text{(A6)}$$

Eq. (A6) shows that the coefficient on the cubic term is invariant to the units of measurement.[2] Whereas, the other coefficients are unit dependent, as shown in Eqs. (A3), (A4), and (A5). This suggests that caution is needed when interpreting such estimated coefficients, given that a switch from positive to negative, for example, could be solely from using a rebased activity variable. Like the quadratic case, the standard errors, and *t-values* (and consequently the significances) of the non-leading coefficients would also change with the rebased data, while

[2] This also holds for the coefficients of any other terms in the function that are not involved in a multiplicative term, such as the natural logarithm of energy consumption.

they remain invariant for the leading term coefficient. To show these, the impact of rescaling on the *t-values* of β_1, β_2 and β_3 are considered below:

$$t_{\beta_1} = \frac{\beta_1}{\sqrt{var(\beta_1)}} \tag{A7}$$

$$t_{\beta_2} = \frac{\beta_2}{\sqrt{var(\beta_2)}} \tag{A8}$$

$$t_{\beta_3} = \frac{\beta_3}{\sqrt{var(\beta_3)}} \tag{A9}$$

$$t_{\beta_1^*} = \frac{\beta_1^*}{\sqrt{var(\beta_1^*)}} \tag{A10}$$

$$t_{\beta_2^*} = \frac{\beta_2^*}{\sqrt{var(\beta_2^*)}} \tag{A11}$$

$$t_{\beta_3^*} = \frac{\beta_3^*}{\sqrt{var(\beta_3^*)}} \tag{A12}$$

Considering Eqs. (A4)-(A6) in Eqs. (A10)-(A12) results in the following:

$$t_{\beta_1^*} = \frac{\beta_1 - 2\beta_2 lna + 3\beta_3 ln^2 a}{\sqrt{var(\beta_1 - 2\beta_2 lna + 3\beta_3 ln^2 a)}} =$$

$$= \frac{\beta_1}{\sqrt{var(\beta_1)}} \sqrt{\frac{var(\beta_1)}{var(\beta_1 - 2\beta_2 lna + 3\beta_3 ln^2 a)}}$$

$$-2lna \frac{\beta_2}{\sqrt{var(\beta_2)}} \sqrt{\frac{var(\beta_2)}{var(\beta_1 - 2\beta_2 lna + 3\beta_3 ln^2 a)}}$$

$$+3ln^2 a \frac{\beta_3}{\sqrt{var(\beta_3)}} \sqrt{\frac{var(\beta_3)}{var(\beta_1 - 2\beta_2 lna + 3\beta_3 ln^2 a)}} \tag{A13}$$

$$t_{\beta_2^*} = \frac{\beta_2 - 3\beta_3 lna}{\sqrt{var(\beta_2 - 3\beta_3 lna)}}$$

$$= \frac{\beta_2}{\sqrt{var(\beta_2)}} \sqrt{\frac{var(\beta_2)}{var(\beta_2 - 3\beta_3 lna)}}$$

$$-3lna \frac{\beta_3}{\sqrt{var(\beta_3)}} \sqrt{\frac{var(\beta_3)}{var(\beta_2 - 3\beta_3 lna)}} \quad \text{(A14)}$$

$$t_{\beta_3^*} = \frac{\beta_3}{\sqrt{var(\beta_3)}} \quad \text{(A15)}$$

As a result, a comparison of Eq. (A7) with Eq. (A13), Eq. (A8) with Eq. (A14) and Eq. (A9) with Eq. (A15) gives us:

$$t_{\beta_1} \neq t_{\beta_1^*}, t_{\beta_2} \neq t_{\beta_2^*} \quad (A16)$$

while

$$t_{\beta_3} = t_{\beta_3^*} \quad (A17)$$

Therefore, the *t-values* and hence the statistical significances of the coefficients of the non-leading terms are unit dependent, while the significance of the coefficient of the leading term is invariant to the rescaling. This implies that the only *necessary condition* for a cubic (N-shaped) EKC is that the leading term, β_3 is positive and statistically significant.[3]

A2.1.2 Estimated turning points

The turning point for the cubic logarithmic specifications is found by differentiating and setting equal to zero. For Eq. (2b) with the 'raw' units, this gives:

$$Y^{TP} = \exp\left(\frac{-\beta_2 \pm \sqrt{\beta_2^2 - 3\beta_1\beta_3}}{3\beta_3}\right) \quad \text{(A18)}$$

---

[3] Note, as stated in the main text we are not trying to pretend that this is a new finding as such, but what is new is that it is the *only* necessary condition. For example, Sinha et al. (2018; p. 4) state that the "necessary condition for a cubic (N-shaped) non-logarithmic EKC like Eq. (2b) is that α_1, $\alpha_3 > 0$ and $\alpha_2 < 0$"; however, we are explicitly concerned here with the cubic (N-shaped) logarithmic version of the EKC where, as we show, it is only the leading term, β_3, that is relevant.

but for Eq. (A1) with the re-based units, this gives:

$$Y^{*TP} = \exp\left(\frac{-\beta_2^* \pm \sqrt{\beta_2^{*2} - 3\beta_1^*\beta_3^*}}{3\beta_3^*}\right) \tag{A19}$$

However, substituting Eqs. (A3) to (A6) into Eq. (A19) gives:

$$Y^{*TP} = \exp\left(\frac{-\beta_2 + 3\beta_3 lna \pm \sqrt{(\beta_2 - 3\beta_3 lna)^2 - (\beta_1 - 2\beta_2 lna + 3\beta_3 ln^2 a)\beta_3}}{3\beta_3}\right)$$

$$= \exp\left(\frac{-\beta_2 + 3\beta_3 lna \pm \sqrt{\beta_2^2 - 6\beta_2\beta_3 lna + 9\beta_3^2 ln^2 a - 3\beta_1\beta_3 + 6\beta_2\beta_3 lna - 9\beta_3^2 ln^2 a}}{3\beta_3}\right)$$

$$= \exp\left(\frac{-\beta_2 + 3\beta_3 lna \pm \sqrt{\beta_2^2 - 3\beta_1\beta_3}}{3\beta_3}\right) = \exp\left(\frac{-\beta_2 \pm \sqrt{\beta_2^2 - 3\beta_1\beta_3}}{3\beta_3} + lna\right)$$

$$= \exp\left(\frac{-\beta_2 \pm \sqrt{\beta_2^2 - 3\beta_1\beta_3}}{3\beta_3}\right)\exp(lna) = a\exp\left(\frac{-\beta_2 \pm \sqrt{\beta_2^2 - 3\beta_1\beta_3}}{3\beta_3}\right) = aY^{TP} \tag{A20}$$

Therefore, rescaling the variable results in rescaling the turning point by the same number.

A2.1.3 Estimated elasticity and statistical significance

Despite this, the estimated elasticity of E with respect to Y is not unit dependent. The elasticity for Eq. (2b) is given by:

$$\eta = \frac{\partial E_t}{\partial Y_t}\frac{Y_t}{E_t} = \frac{\partial lnE_t}{\partial lnY_t} = \beta_1 + 2\beta_2 lnY_t + 3\beta_3 ln^2 Y_t \tag{A21}$$

And the elasticity for Eq. (A1) is given by:

$$\eta^* = \frac{\partial E_t}{\partial Y_t^*}\frac{Y_t^*}{E_t} = \frac{\partial lnE_t}{\partial lnY_t^*} = \beta_1^* + 2\beta_2^* lnY_t^* + 3\beta_3^* ln^2 Y_t^* \tag{A22}$$

However, substituting Eq. (2b) and Eqs. (A3) – (A6) into Eq. (A22) and rearranging gives:

$$\eta^* = \beta_1 + 2\beta_2 lnY_t + 3\beta_3 ln^2 Y_t \tag{A23}$$

thus:

$$\eta^* = \eta \tag{A24}$$

so that the estimated elasticity of E with respect to Y is invariant to the units used for the activity variable. The significance of the elasticity for the cubic case is also unit independent, like the quadratic case, as shown by the proof below.

The variance of η is as follow:

$$var(\eta) = var(\beta_1 + 2\beta_2 lnY_t + 3\beta_3 ln^2 Y_t) = var(\beta_1) + 4ln^2 Y_t var(\beta_2) + 9ln^4 Y_t var(\beta_3) + 4lnY_t cov(\beta_1, \beta_2) + 6ln^2 Y_t cov(\beta_1, \beta_3) + 12ln^3 Y_t cov(\beta_2, \beta_3) \tag{A25}$$

Likewise, the variance for η^* is given by:

$$var(\eta^*) = var(\beta_1^* + 2\beta_2^* \ln Y_t^* + 3\beta_3^* ln^2 Y_t^*) \qquad (A26)$$

Considering rescaling and variance-covariance properties, we get the following:

$$var(\eta^*) = var(\beta_1^* + 2\beta_2^* \ln Y_t^* + 3\beta_3^* ln^2 Y_t^*) = var(\beta_1^*) + 4ln^2 Y_t^* var(\beta_2^*) + 9ln^4 Y_t^* var(\beta_3^*) + 4 \ln Y_t^* cov(\beta_1^*, \beta_2^*) + 6ln^2 Y_t^* cov(\beta_1^*, \beta_3^*) + 12ln^3 Y_t^* cov(\beta_2^*, \beta_3^*) = var(\beta_1 - 2\beta_2 lna + 3\beta_3 ln^2 a) + (4ln^2 a + 8lnalnY + 4ln^2 Y) var(\beta_2 - 3\beta_3 lna) + 9(lna + lnY)^4 var(\beta_3) + 4(lna + lnY) cov(\beta_1 - 2\beta_2 lna + 3\beta_3 ln^2 a, \beta_2 - 3\beta_3 lna) + (6ln^2 a + 12lnalnY + 6ln^2 Y) cov(\beta_1 - 2\beta_2 lna + 3\beta_3 ln^2 a, \beta_3) + 12(lna + lnY)^3 cov(\beta_2 - 3\beta_3 lna, \beta_3) = var(\beta_1) + 4ln^2 avar(\beta_2) + 9ln^4 Y_t var(\beta_3) - 4lnacov(\beta_1, \beta_2) + 6ln^2 acov(\beta_1, \beta_3) - 12ln^3 acov(\beta_2, \beta_3) + (4ln^2 a + 8lnalnY + 4ln^2 Y)\big(var(\beta_2) + 9ln^2 avar(\beta_3) - 6lnacov(\beta_2, \beta_3)\big) + 9(ln^4 a + 4ln^3 alnY + 6ln^2 aln^2 Y + 4lnaln^3 Y + ln^4 Y) var(\beta_3) + (4lna + 4lnY))(cov(\beta_1, \beta_2) - 3lnacov(\beta_1, \beta_3) - 2lnavar(\beta_2) + 6ln^2 acov(\beta_2, \beta_3) + 3ln^2 acov(\beta_2, \beta_3) - 9ln^3 avar(\beta_3)) + (6ln^2 a + 12lnalnY + 6ln^2 Y)(cov(\beta_1, \beta_3) - 2lnacov(\beta_2, \beta_3) + 3ln^2 avar(\beta_3)) + 12(ln^3 a + 3ln^2 alnY + 3lnaln^2 Y + ln^3 Y)(cov(\beta_2, \beta_3) - 3lnavar(\beta_3)) = var(\beta_1) + (4ln^2 a + 4ln^2 a + 8lnalnY + 4ln^2 Y - 8ln^2 a - 8lnalnY) var(\beta_2) + (9ln^4 a + 36ln^4 a + 72ln^3 alnY + 36ln^2 aln^2 Y + 9ln^4 a + 36ln^3 alnY + 54ln^2 aln^2 Y + 36lnaln^3 Y + 9ln^4 a - 36ln^4 a - 36ln^3 alnY + 18ln^4 a + 36ln^3 alnY + 18ln^2 aln^2 Y - 36ln^4 a - 108ln^3 alnY - 108ln^2 aln^2 Y - 36lnaln^3 Y) var(\beta_3)) + (-4lna + 4lna + 4lnY)) cov(\beta_1, \beta_2) + (6ln^2 a - 12ln^2 a - 12lnalnY + 6ln^2 a + 12lnalnY + 6ln^2 Y) cov(\beta_1, \beta_3) + (-12ln^3 a - 24ln^3 a - 48ln^2 alnY - 24lnaln^2 Y + 36ln^3 a + 36ln^2 alnY - 12ln^3 a - 24ln^2 alnY - 12lnaln^2 Y + 12ln^3 a + 36ln^2 alnY + 36lnaln^2 Y + 12ln^3 Y) cov(\beta_2, \beta_3) = var(\beta_1) + 4ln^2 Y_t var(\beta_2) + 9ln^4 Y_t var(\beta_3) + 4lnY_t cov(\beta_1, \beta_2) + 6ln^2 Y_t cov(\beta_1, \beta_3) + 12ln^3 Y_t cov(\beta_2, \beta_3) = var(\eta) \qquad (A27)$$

Thus, in the cubic case, like the quadratic case, the elasticity estimates and their significance levels are unit independent.

A2.1.4 Sufficient condition

Mathematically, the sufficient condition for a cubic (N-shaped) EKC like Eq. (2b) is that the discriminant of the derivative function is positive, that is $\beta_2^2 - 3\beta_1\beta_3 > 0$ with $\beta_3 > 0$ (and statistically significant) and the turning points are within the sample range. In other words, the sufficient condition for an estimated cubic (N-shaped) EKC is that the turning point is within the sample range with the estimated pairwise elasticities positive and significant for the initial upward sloping part of the estimated curve, but they approach zero and become insignificant at the first turning point, thereafter, becoming negative and significant on the downward sloping part. And that after the first turning point, the estimated pairwise elasticities continue to be negative and significant but approach zero and become insignificant at the second turning point, thereafter, becoming positive again and significant on the next upward sloping part of the estimated curve.

A2.2 Quartic EKC Specification

A2.2.1 Estimated parameters and statistical significance

Utilising the same scaling as in Eq. (3), Eq. (2c) becomes:

$$lnE_t = \beta_0^* + \beta_1^* lnY_t^* + \beta_2^* ln^2Y_t^* + \beta_3^* ln^3Y_t^* + \beta_4^* ln^4Y_t^* \tag{A28}$$

and substituting Eq. (3) into Eq. (A28) and rearranging gives:

$$lnE_t = [\beta_0^* + \beta_1^* lna + \beta_2^* ln^2a + \beta_3^* ln^3a] + [\beta_1^* + 2\beta_2^* lna + 3\beta_3^* ln^2a + 4\beta_4^* ln^3a]lnY_t + [\beta_2^* + 3\beta_3^* lna + 6\beta_4^* ln^2a]ln^2Y_t + [\beta_3^* + 4\beta_4^* lna]ln^3Y_t + \beta_4^* ln^4Y_t \tag{A29}$$

Given the left-hand side of Eqs. (2c) and (A29) are the same, the two equations can be equated to give the following formulas relating the new to the original coefficients:

$$\beta_0^* = \beta_0 - \beta_1 lna + \beta_2 ln^2a - \beta_3 ln^3a + \beta_4 ln^4a \tag{A30}$$

$$\beta_1^* = \beta_1 - 2\beta_2 lna + 3\beta_3 ln^2a - 4\beta_4 ln^3a \tag{A31}$$

$$\beta_2^* = \beta_2 - 3\beta_3 lna + 6\beta_4 ln^2 a \quad \text{(A32)}$$

$$\beta_3^* = \beta_3 - 4\beta_4 lna \quad \text{(A33)}$$

$$\beta_4^* = \beta_4 \quad \text{(A34)}$$

As Eq. (A34) shows the coefficient on the fourth order term is invariant to the units of measurement. However, the other coefficients are unit dependent, as shown in Eqs. (A31), (A32), and (A33). The related *t-values* can be compared, like with the quadratic and cubic cases, to see the impact of scaling on the statistical significance of the corresponding coefficients. This implies that the only *necessary* condition for a quartic (M-shaped) EKC is that the leading term, β_4 is negative and statistically significant.[4]

A2.2.2 Estimated turning points

The formula for turning points can be derived equating the derivative of (2c) to zero, which gives the cubic equation below:

$$4\beta_4 ln^3 Y_t + 3\beta_3 ln^2 Y_t + 2\beta_2 lnY_t + \beta_1 = 0 \quad \text{(A35)}$$

Eq. (A35) can be solved using the so-called Cardano's formula (see, Spiegel, 1968, *inter alia*).

$$Y^{TP} = \exp\left(\sqrt[3]{-\frac{q}{2} + \sqrt{\frac{q^2}{4} + \frac{p^2}{27}}} + \sqrt[3]{-\frac{q}{2} - \sqrt{\frac{q^2}{4} + \frac{p^2}{27}}}\right) \quad \text{(A36)}$$

Where,

$$p = \frac{8\beta_2\beta_4 - 3\beta_3^2}{16\beta_4^2} \text{ and } q = \frac{1}{32}\frac{\beta_3{}^3}{\beta_4{}^3} - \frac{1}{8}\frac{\beta_2\beta_3}{\beta_4^2} + \frac{\beta_1}{4\beta_4} \quad \text{(A37)}$$

Since the formulas are highly involved, we will not derive the relationship between the two cases. Rather, we will derive the effect of rescaling done using software and provided in the empirical section.

---

[4] Again, we are not trying to pretend that this is a new finding as such, but what is new is that it is the *only* necessary condition i.e., for a quartic (M-shaped) logarithmic version of the EKC it is only the leading term, β_3, that is relevant.

A2.2.3 Estimated elasticity and statistical significance

Despite this, the estimated elasticity of E with respect to Y is not unit dependent. The elasticity for Eq. (2c) is given by:

$$\eta = \frac{\partial E_t}{\partial Y_t}\frac{Y_t}{E_t} = \frac{\partial lnE_t}{\partial lnY_t} = \beta_1 + 2\beta_2 lnY_t + 3\beta_3 ln^2Y_t + 4\beta_4 ln^3Y_t \qquad (A38)$$

And the elasticity for Eq. (A28) is given by:

$$\eta^* = \frac{\partial E_t}{\partial Y_t^*}\frac{Y_t^*}{E_t} = \frac{\partial lnE_t}{\partial lnY_t^*} = \beta_1^* + 2\beta_2^* lnY_t^* + 3\beta_3^* ln^2Y_t^* + 4\beta_4^* ln^3Y_t^* \qquad (A39)$$

However, substituting Eq. (2c) and Eqs. (A30) – (A34) into Eq. (A39) and rearranging gives:

$$\eta^* = \beta_1 + 2\beta_2 lnY_t + 3\beta_3 ln^2Y_t + 4\beta_4 ln^3Y_t \qquad (A40)$$

thus:

$$\eta^* = \eta \qquad (A41)$$

The variance exercises are straightforward, like the quadratic and cubic cases, and are briefly given below. The variance of the elasticity can be expressed as follows:

$$var(\eta) = var(\beta_1 + 2\beta_2 lnY_t + 3\beta_3 ln^2Y_t + 4\beta_4 ln^3Y_t) = var(\beta_1) + 4ln^2Y_t * var(\beta_2) + 9ln^4Y_t * var(\beta_3) + 16ln^6Y_t * var(\beta_4)$$
$$+4lnY_t * cov(\beta_1, \beta_2) + 6ln^2Y_t * cov(\beta_1, \beta_3) + 8ln^3Y_t * cov(\beta_1, \beta_4) + 12ln^3Y_t * cov(\beta_2, \beta_3) + 16ln^4Y_t * cov(\beta_2, \beta_4) + 24ln^5Y_t * cov(\beta_3, \beta_4) (A42)$$

Then the variance of (A40) will be:

$$var(\eta^*) = var(\beta_1^* + 2\beta_2^* lnY_t^* + 3\beta_3^* ln^2Y_t^* + 4\beta_4^* ln^3Y_t^*) = var(\beta_1^*) + 4ln^2Y_t^* * var(\beta_2^*) + 9ln^4Y_t^* * var(\beta_3^*) + 16ln^6Y_t^* * var(\beta_4^*)$$
$$+4lnY_t * cov(\beta_1^*, \beta_2^*) + 6ln^2Y_t^* * cov(\beta_1^*, \beta_3^*) + 8ln^3Y_t^* * cov(\beta_1^*, \beta_4^*) + 12ln^3Y_t^* * cov(\beta_2^*, \beta_3^*) + 16ln^4Y_t^* * cov(\beta_2^*, \beta_4^*) + 24ln^5Y_t^* * cov(\beta_3^*, \beta_4^*) (A43)$$

Considering the properties of variance and covariances, and using properties of logarithmic functions and expansion of binomials, it can be shown that the right-hand side of (A43) can be expressed as below:

$$
\begin{aligned}
&var(\eta^*) \\
&= var(\beta_1) + 4ln^2a * var(\beta_2) + 9ln^4a * var(\beta_3) + 16ln^6a * var(\beta_4) + (-4lna + 4lna + 4lny) * cov(\beta_1, \beta_2) \\
&+ (6ln^2a + ln^2a + 12lnalny + ln^2y - 12ln^2a - 12lnalny) * cov(\beta_1, \beta_3) \\
&+ (-8ln^3a - 24ln^3a - 48ln^3alny - 24lnaln^2Y_t + 24ln^3a + 24ln^2alny + 8ln^3a + 24ln^2alny + 24lnaln^2Y_t + 8ln^3Y_t) * cov(\beta_1, \beta_4) \\
&+ (-12ln^3a - 36ln^3a - 72ln^2alnY - 36lnaln^2Y_t + 36ln^3a + 36ln^2alny + 12ln^3a + 36ln^2alny + 36lnaln^2Y_t + 12ln^3Y_t) * cov(\beta_2, \beta_3) \\
&+ (16ln^4a + 96ln^4a + 192ln^3alny + 96ln^2aln^2Y_t + 16ln^4a + 64ln^3alnY_t + 96ln^2aln^2Y_t + 64lnaln^3Y_t + 16ln^4Y_t - 64ln^4a - 64lnaln^3Y_t - 64ln^4a \\
&- 192ln^3alnY_t - 192ln^2aln^2Y_t - 64lnaln^3Y_t) * cov(\beta_2, \beta_4) \\
&+ (-24ln^5a - 240ln^5a - 480ln^4alnY_t - 240ln^3aln^2Y_t - 120ln^5a - 480ln^4alnY_t - 720ln^3aln^2Y_t - 480ln^2aln^3Y_t - 120lnaln^4Y_t + 120ln^5a \\
&+ 120ln^4alnY_t + 240ln^5a + 720ln^4alnY_t + 720ln^3aln^2Y_t + 240ln^2aln^3Y_t + 24ln^5a + 120ln^4alnY_t + 240ln^3aln^2Y_t + 240ln^2aln^3Y_t + 120ln^4alnY_t \\
&+ 24ln^5a) * cov(\beta_3, \beta_4) + (4ln^2a + 8lnalnY_t + 4ln^2Y_t - 8ln^2a - 8lnalnY_t) * var(\beta_2) \\
&+ (54ln^4a + 108ln^3alnY_t + 54ln^2aln^2Y_t + 9ln^4a + 36ln^3alnY_t + 54ln^2aln^2Y_t + 36lnaln^3Y_t + 9ln^4a - 36ln^4a - 36ln^3alnY_t - 36ln^4a - 108ln^3alnY_t \\
&- 108ln^2aln^2Y_t - 36lnaln^3Y_t) * var(\beta_3) \\
&+ (240ln^6a + 480ln^5alnY_t + 240ln^4aln^2Y_t + 240ln^6a + 960ln^5alnY_t + 1440ln^4aln^2Y_t + 960ln^3aln^3Y_t + 240ln^2aln^4Y_t + 16ln^6a + 96ln^5alnY_t \\
&+ 240ln^4aln^2Y_t + 320ln^3aln^3Y_t + 240ln^2aln^4Y_t + 96lnaln^5Y_t + 160ln^6Y_t - 96ln^6a - 96ln^5alnY_t - 320ln^6a - 960ln^5alnY_t - 960ln^4aln^2Y_t \\
&- 320ln^3aln^3Y_t - 96ln^6a - 480ln^5alnY_t - 960ln^4aln^2Y_t - 960ln^3aln^3Y_t - 480ln^2aln^4Y_t - 96lnaln^5Y_t) * var(\beta_4) \\
&= \begin{matrix} = var(\beta_1) + 4ln^2Y_t * var(\beta_2) + 9ln^4Y_t * var(\beta_3) + 16ln^6Y_t * var(\beta_4) \\ +4lnY_t * cov(\beta_1, \beta_2) + 6ln^2Y_t * cov(\beta_1, \beta_3) + 8ln^3Y_t * cov(\beta_1, \beta_4) + 12ln^3Y_t * cov(\beta_2, \beta_3) + 16ln^4Y_t * cov(\beta_2, \beta_4) + 24ln^5Y_t * cov(\beta_3, \beta_4) = var(\eta) \end{matrix} \text{(A44)}
\end{aligned}
$$

Like quadratic and cubic cases, Eqs. (A41) and Eq. (A44) show that the elasticity estimates and their variances are not affected by the rescaling. In other words, their standard errors, *t-values*, and significance levels are all *not unit dependent*.

A2.2.4 Sufficient condition

Mathematically, the sufficient condition for a quartic (M-shaped) EKC like Eq. (2c) is that the discriminant of the derivative function is negative,[5] that is $\frac{q^2}{4} + \frac{p^3}{27} < 0$ [6] with $\beta_4 < 0$ (and statistically significant), and the turning points are within the sample range. In other words, the sufficient condition for an estimated quartic (M-shaped) EKC as in Eq. (2c) is that the turning point is within the sample range with the estimated pairwise elasticities positive and significant for the initial upward sloping part of the estimated curve but approach zero and become insignificant at the first turning point, thereafter, becoming negative and significant on the downward sloping part. That after the first turning point the estimated pairwise elasticities continue to be negative and significant but approach zero and become insignificant at the second turning point, thereafter, becoming positive again and significant on the next upward

---

[5] See, inter alia, Kichenassamy (2015) for details of the different solution techniques of cubic equations.

[6] Where q and p are defined in Eq. (A37) above.

sloping part of the estimated curve. And that the estimated pairwise elasticities continue to be positive and significant after the second turning point, but they approach zero and become insignificant at the third turning point, thereafter becoming negative again and significant on the next downward sloping part of the estimated curve.

Additional references in on-line annex that do not appear in the main text

Enders W. 2015. Applied Econometrics Time Series, Wiley Series in Probability and Statistics University of Alabama, Tuscaloosa, AL, USA.

Engle, Robert F & Granger, Clive W J, 1987. Co-integration and Error Correction: Representation, Estimation, and Testing. Econometrica, 55(2), 251-276.

Kichenassamy S. Continued proportions and Tartaglia's solution of cubic equations. 2015. Historia Mathematica 42(4), 407-435.

Spiegel, M. R. 1968 Mathematical Handbook of Formulas and Tables. McGraw-Hill.
